# Diagnostic Yield and Clinical Actionability of Genetic Testing in Neurodevelopmental Disorders: A Systematic Review and Meta-Analysis

**DOI:** 10.3390/ijms27209113

**Published:** 2026-10-14

**Authors:** Alexandra Aurora Dumitra, Ioana Streață, Adina Turcu-Știolică, Anca Lelia Riza, Rebecca Cristiana Șerban, Mihai Gabriel Cucu, Alexandru Mihai Prică, Florin Burada

**Affiliations:** 1 Doctoral School, University of Medicine and Pharmacy of Craiova, 200349 Craiova, Romaniaalexandru.prica@umfcv.ro (A.M.P.); 2 Regional Centre of Medical Genetics Dolj, Emergency County Hospital Craiova, 200642 Craiova, Romania; 3 Human Genomics Laboratory, Research Center for Gastroenterology and Hepatology (CCGH), University of Medicine and Pharmacy of Craiova, 200638 Craiova, Romania; 4 Department of Pharmacoeconomics, Faculty of Pharmacy, University of Medicine and Pharmacy of Craiova, 200349 Craiova, Romania

**Keywords:** neurodevelopmental disorders, autism spectrum disorder, genetic testing, diagnostic yield, clinical actionability, whole-exome sequencing, chromosomal microarray

## Abstract

Genetic testing is increasingly central to the evaluation of neurodevelopmental disorders (NDDs), yet most evidence emphasises diagnostic yield rather than whether findings change clinical care. We conducted a systematic review and meta-analysis (Preferred Reporting Items for Systematic Reviews and Meta-Analyses [PRISMA] 2020) of genetic testing across the diagnostic pathway in NDD populations, focusing on autism spectrum disorder (ASD), intellectual disability, and global developmental delay, to quantify pooled diagnostic yield and clinical actionability. A systematic search of PubMed and Web of Science, with an expanded population and outcome term set, was completed on 1 October 2026, supplemented by an artificial intelligence (AI)-assisted search (Undermind) and reference-list screening. Twenty-three studies were included in a qualitative synthesis and 22 in a quantitative meta-analysis of diagnostic yield (N = 7641 patients tested; diagnostic-yield analysis comprised 23 data points, as Charouf et al. contributed separate whole-exome sequencing (WES) and whole-genome sequencing (WGS) cohorts). Single-proportion random-effects meta-analysis with the Freeman–Tukey double arcsine transformation produced a pooled diagnostic yield of 28% (95% confidence interval [CI] 21–35%; I^2^ = 96.1%) and pooled clinical actionability of 58% (95% CI 44–72%; I^2^ = 95.7%) across 16 studies (N = 1538 patients with confirmed diagnoses). A significant yield gradient was observed across modalities (chromosomal microarray analysis [CMA], 16%; WES, 36%; WGS, 43%; *p* < 0.0001). Actionability estimates were robust across sensitivity analyses (54–64%). Genetic testing in NDDs functions not only as an etiological tool but as an input to personalised management, including pharmacological optimisation, comorbidity surveillance, and resolution of the diagnostic odyssey, albeit with very low certainty of evidence per Grading of Recommendations, Assessment, Development and Evaluations (GRADE), reflecting substantial heterogeneity and observational study design.

## 1. Introduction

Neurodevelopmental disorders (NDDs) comprise a heterogeneous group of conditions—including autism spectrum disorder (ASD), attention-deficit/hyperactivity disorder (ADHD), intellectual disability (ID), and global developmental delay (GDD)—whose boundaries frequently overlap and whose reported prevalence has risen in recent years [1,2,3]. This review focuses on the broader NDD spectrum, with particular attention to ASD, ID, and GDD, which constitute the primary evidence base identified through systematic search; although ADHD and language delay were included in the original eligibility criteria, no study meeting inclusion criteria reported yield or actionability data for a primary ADHD or isolated language-delay cohort, narrowing the final evidence base accordingly. Although the aetiology of these conditions is multifactorial (combining genetic, neurological, metabolic, perinatal, and environmental factors), necessitating integrated, multidisciplinary care models, genetics plays a central and increasingly well-characterised role [1,4,5].

For GDD/ID, genetic abnormalities account for up to half of cases, with reported diagnostic yields ranging widely from 20% to 80% depending on phenotypic severity and the presence of neurological or congenital features [5,6,7,8,9]. ASD is among the most heritable neurodevelopmental conditions, with heritability estimates of 64–91% [10,11], reflecting the combined contribution of de novo and inherited genetic variants [12] and motivating targeted clinical genetic testing strategies in this population [13,14]; nonetheless, historical diagnostic yields were modest (6–15%), and ambiguous results or variants of uncertain significance (VUS) continue to complicate counselling [15,16,17,18]. Identifying an underlying genetic cause carries tangible benefits, enabling more accurate prognostication and better-informed family planning and long-term care [19,20].

Diagnostic strategies have evolved accordingly. Chromosomal microarray (CMA) replaced karyotyping as the first-tier test for developmental delay (DD)/ID, ASD, and multiple congenital anomalies in 2010, with a 15–20% yield [21,22]. Whole-exome sequencing (WES)—particularly trio-based WES for the detection of de novo variants—is now regarded as the most informative first-tier test for NDDs, and next-generation sequencing (NGS) detects rare variants in up to 30% of the ASD population when initial testing is negative [15,23,24,25,26,27,28,29,30,31,32]. Despite this, integration of genomic data into routine clinical care remains uneven, and the frequency with which results actually alter therapeutic decisions is poorly represented in the literature [22].

This evidence gap is the central motivation for the present work. The majority of genetic studies in ASD and NDDs emphasise “yield” rather than whether findings affect decisions about healthcare, education, or reproduction, and prior reviews have noted that only a subset of studies report long-term outcomes, cost-efficiency, or concrete management change [30,31,32,33,34,35,36,37]. Where prospective data exists, pathogenic findings frequently prompt new surveillance, referrals, or treatment modifications, demonstrating the feasibility of translating molecular results into clinical action [22,23,27,38].

To address this gap, we conducted a systematic review and meta-analysis quantifying both pooled diagnostic yield and clinical actionability across published NDD studies, with sensitivity analyses by testing modality and pre-specified subgroups. Our primary research question examines the extent to which genetic testing across the diagnostic pathway yields clinically actionable outcomes across the NDD spectrum—specifically through optimised medical monitoring and personalised therapeutic management—as measured by diagnostic yield, pharmacological modifications, and specialist referral rates. Because none of the included studies contained an untreated control group, the comparison component of the Population, Intervention, Comparison, Outcome (PICO) framework is addressed through the reported rate of clinical management changes following a genetic diagnosis, rather than by direct comparison with untested groups.

## 2. Materials and Methods

### 2.1. Search Strategy

Systematic searches of PubMed (National Library of Medicine, Bethesda, MD, USA; https://pubmed.ncbi.nlm.nih.gov/) and Web of Science (Clarivate, London, UK; https://www.webofscience.com/) were completed on 1 October 2026 (date of access for both databases), using Boolean operators (AND, OR) and free-text terms across three concept blocks: intervention (“genetic testing” OR “genomic testing” OR “WES” OR “Whole Exome Sequencing” OR “exome sequencing” OR “genome sequencing” OR “whole genome sequencing” OR “WGS” OR “microarray”), population (“autism” OR “ASD” OR “Autism Spectrum Disorder” OR “ADHD” OR “Attention-Deficit/Hyperactivity Disorder” OR “language delay” OR “Neurodevelopmental Disorders” OR “NDD” OR “intellectual disability” OR “developmental delay” OR “global developmental delay”), and outcome (“personalized medicine” OR “individualized therapy” OR “clinical management” OR “therapeutic management” OR “clinical utility” OR “clinical impact” OR “actionability”). A publication-date filter (20 November 2015 onward) was applied to both database searches. The searches were supplemented by an artificial intelligence AI-assisted literature search (Undermind, Undermind, San Francisco, CA, USA; https://www.undermind.ai/; accessed on 1 October 2026) and by screening of reference lists of included articles, neither of which was date-restricted, which accounts for the inclusion of several studies published before the database filter’s start date. Reporting followed the Preferred Reporting Items for Systematic Reviews and Meta-Analyses (PRISMA) 2020 guidelines [39]. This systematic review was registered with the International Prospective Register of Systematic Reviews (PROSPERO; CRD420261452265; https://www.crd.york.ac.uk/PROSPERO/view/CRD420261452265 (accessed on 2 October 2026); PROSPERO’s registered population criteria (ASD, ID, DD, and GDD) are consistent with the PICO framework applied throughout this review (Section 2.2). The full, verbatim search strategy, including exact syntax, filters, and date executed, is provided in Supplementary Table S5.

**Figure 1:**
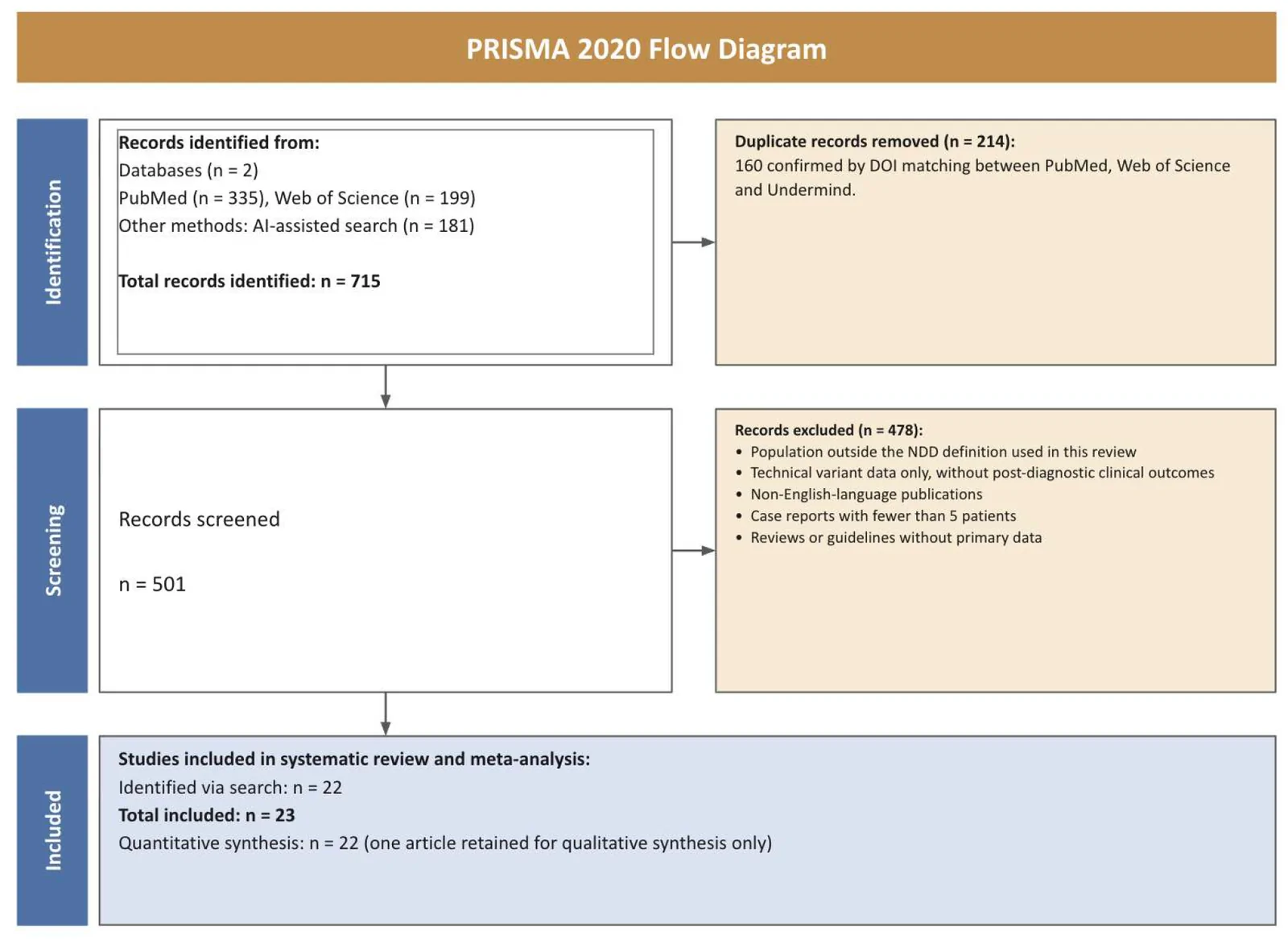
PRISMA flow.

Together with the supplementary routes described above, this search identified the full set of studies included in this review, as detailed in Section 3.1. Full identification, screening, and inclusion counts, including reasons for exclusion, are summarised in Figure 1 and Supplementary Table S6.

### 2.2. Inclusion Criteria, Data Extraction, and Outcomes

Eligibility was structured using the PICO framework. Population: patients with NDDs, including ASD, ADHD, language delay, intellectual disability, developmental delay, and global developmental delay. Intervention: genetic testing. Comparison: standard clinical/therapeutic management without personalised genetic testing. Outcome: personalisation of clinical/therapeutic management (diagnosis, therapeutic interventions, and monitoring), operationalised as clinical actionability rather than demonstrated improvement in long-term patient outcomes (Section 2.4). “Language delay” was operationalised broadly to include developmental language disorder and isolated expressive or receptive delay, in accordance with Diagnostic and Statistical Manual of Mental Disorders, 5th edition (DSM-5) criteria. Studies were excluded if they reported only technical variant data without post-diagnostic clinical data, were published in languages other than English, were case reports with n < 5 (a conventional threshold distinguishing case reports from small case series in systematic reviews), were reviews or guidelines without primary data, or enrolled a population outside the NDD definition used in this review (e.g., epilepsy-only cohorts without comorbid ID, ASD, or GDD). Data were systematically extracted across study identification, methodology, context and population, technical data, and synthesis categories. Title/abstract screening, full-text eligibility assessment, and data extraction were performed by one investigator using a standardised extraction form, with a second investigator independently verifying accuracy for all included studies; discrepancies were resolved through discussion, with a third investigator consulted where consensus could not be reached. The terms “positive finding,” “confirmed diagnosis,” and, where applicable, “clinically relevant genomic finding” are used synonymously throughout to denote a pathogenic or likely pathogenic result meeting the diagnostic-yield definition in Section 2.4, except where an alternative framework (e.g., Selvanayagam et al., 2025 [17]) is explicitly noted.

### 2.3. Study-Level Eligibility Decisions

Detailed study-level eligibility decisions, including the rationale for including four developmental and epileptic encephalopathy (DEE)-associated testing arms (Demos et al., 2019 [40]; Minardi et al., 2020 [41]; Charouf et al., 2024 [42]; WES and whole-genome sequencing (WGS) arms), the exclusion of Pagan et al. (2017) [43] from quantitative synthesis, and the inclusion of six studies in the diagnostic-yield analysis only (Ji et al., 2019 [44]; Wu et al., 2023 [45]; Hakizimana et al., 2026 [46]; Abarca-Barriga et al., 2025 [47]; Monroe et al., 2016 [48]; Ilic et al., 2024 [49]), are summarised below. DEE-associated cohorts were retained because their monogenic aetiology drives neurodevelopmental impairment irrespective of seizures, because of substantial genetic and phenotypic overlap with ASD and ID (e.g., *SCN1A*, *MECP2*, *KCNQ2*, *STXBP1*), and because current American College of Medical Genetics and Genomics (ACMG) guidance recommends exome/genome sequencing for ID/DD without restriction by seizure comorbidity [2,36,50,51,52]. Sequencing cohorts focused on epilepsy likewise report diagnostic genetic findings in both children and adults [53,54,55] (these studies were excluded from the synthesis; Supplementary Table S6). Pagan et al. (2017) [43] was retained for qualitative synthesis only, as their case–control biochemical design did not yield a valid diagnostic-yield denominator compatible with the effect measure used across the other included studies (no significant *AANAT/ASMT* enrichment was observed, *p* = 0.56 vs. controls) and no genotype-directed management changes were recorded. The six studies listed above were included in the yield analysis only, as none reported a formal actionability fraction with a valid denominator. Charouf et al. (2024) [42] combined WES and WGS testing within a single cohort; because clinical actionability was reported only as a combined figure across both modalities, this study was retained in the primary actionability analysis but excluded from the modality-stratified actionability subgroup analysis (Section 3.4.3). For this study, actionability was operationalised as aetiology-specific treatment changes (anti-seizure medication or dietary therapy; 6 of 31 diagnosed children), a conservative estimate that excludes the specialist referrals also reported (15 patients). Nakhleh Francis et al. (2023) [56] reported actionability across four non-mutually exclusive categories (active management, surveillance, family-focused outcomes, specialist referral) without a combined “any action” figure; the specialist-referral category was used as the primary actionability estimate for this study. The impact of these decisions was evaluated through pre-specified and post hoc sensitivity analyses (Section 2.5).

### 2.4. Definition and Ascertainment of Outcomes

Diagnostic yield was defined as the proportion of tested patients receiving a confirmed pathogenic or likely pathogenic genetic diagnosis. This definition was applied consistently across the 23 included studies, with the exception of Selvanayagam et al. (2025) [17], whose reported figure of 49.5% reflects the proportion of families receiving any clinically relevant genomic finding under a research return-of-results framework; this exception was evaluated through a sensitivity analysis restricted to “flag = 1” studies, defined a priori as studies using standard, directly comparable outcome definitions and denominators, as opposed to “flag = 2” studies with non-standard ascertainment methods, broader outcome frameworks, or incomplete denominators (Supplementary Table S1). Clinical actionability was defined as the proportion of patients with a confirmed genetic diagnosis who received at least one specific management recommendation directly attributable to that finding-specialist referral, targeted investigation, initiation or contraindication of medication, surgical or procedural intervention, or structured surveillance, consistent with prior frameworks in genomic medicine [19,36,37]. Studies with non-standard denominator structures or ascertainment methods (Jang et al., 2019 [22]; Saam et al., 2008 [57]; Henderson et al., 2014 [58]; Nakhleh Francis et al., 2023 [56]) and the broader three-category framework of Selvanayagam et al. (2025) [17] were identified a priori. Jang et al. (2019) [22] reported actionability across all variant-pathogenicity classes (581/617 with documented follow-up); Saam et al. (2008) [57] ascertained actionability via physician telephone interview (responses obtained for 48 of 87 patients with a positive result, 55% response rate); Henderson et al. (2014) [58] restricted the denominator to patients with documented electronic medical record (EMR) follow-up (187/227); and Nakhleh Francis et al. (2023) [56] reported actionability across four non-aggregable categories, with the specialist-referral category (20/74) used as the primary estimate (Section 2.3). Their collective impact, together with the broader three-category actionability framework of Selvanayagam et al. (2025) [17], was assessed through pre-specified sensitivity analyses restricted to methodologically homogeneous studies (n = 9 for actionability, n = 15 for diagnostic yield).

### 2.5. Statistical Analysis

Quantitative synthesis was performed separately for diagnostic yield and clinical actionability using meta-analysis of single proportions. For each study, the confirmed diagnoses or actionable cases and the total patients tested or diagnosed were extracted directly from the original publication; no summary statistics were missing for the primary analyses. Comparative effect-size estimation was not possible, as no included study contained an untreated control group. Analyses were performed in R (v4.5.3; R Foundation for Statistical Computing, Vienna, Austria; https://www.R-project.org/, accessed on 2 October 2026) using the meta package (v8.5.0; Comprehensive R Archive Network, https://CRAN.R-project.org/package=meta, accessed on 2 October 2026) [59]. The metaprop() function was used with the Freeman–Tukey double arcsine transformation (sm = “PFT”), selected to stabilise the variance of proportions approaching 0% or 100% [60]. A random-effects model was specified a priori, given substantial differences in population, setting, diagnostic criteria, and testing era; between-study variance (tau^2^) was estimated by the restricted maximum likelihood (REML) method. Heterogeneity was quantified using I^2^, the Cochran Q statistic, and tau^2^. Pre-specified subgroup analyses were conducted by modality (CMA, WES, WGS), with between-subgroup differences evaluated by the Q-between test. Sensitivity analyses included restriction to methodologically homogeneous studies (flag = 1); exclusion of Selvanayagam et al. (2025) [17] from the actionability analysis; exclusion of epilepsy/DEE-associated cohorts; and exclusion of Nakhleh Francis et al. (2023) [56] from the actionability analysis given its non-aggregable actionability definition (Section 2.3). Analyses using logit-transformed proportions and a generalised linear mixed model (GLMM; method.tau = “ML”) were additionally performed to assess robustness to the choice of variance-stabilising transformation, alongside 95% prediction intervals to characterise the range of effects expected in a new clinical setting given the high observed heterogeneity. Publication bias was assessed visually with funnel plots and formally with Egger’s regression test (threshold *p* < 0.1) [61]. Analyses were conducted separately for diagnostic yield (23 data points from 22 studies, N = 7641) and clinical actionability (16 studies, N = 1538).

The overall certainty of the body of evidence for each outcome was assessed using the Grading of Recommendations, Assessment, Development and Evaluations (GRADE) approach [62], adapted for single-group prevalence/proportion meta-analyses in the absence of a comparator group (Supplementary Table S4).

### 2.6. Methodological Quality Assessment

Methodological quality was assessed using the Joanna Briggs Institute (JBI) Critical Appraisal Checklist for Studies Reporting Prevalence Data (nine items; scored Yes/No/Unclear). Studies scoring ≥7 were classified as high quality, 5–6 as moderate, and <5 as low. The JBI checklist was preferred over the Newcastle–Ottawa Scale, which is designed for comparative cohort and case–control studies and is inappropriate for single-arm proportion studies without an untreated comparator. Quality appraisal using the JBI checklist was performed by one investigator, with a second investigator independently verifying the scores for all included studies; discrepancies were resolved by discussion, with a third investigator consulted where consensus could not be reached.

## 3. Results

A pooled diagnostic yield of 28% (95% confidence interval [CI] 21–35%; I^2^ = 96.1%) was identified across 23 data points from 22 studies (N = 7641 patients tested). Clinical actionability was documented in 58% (95% CI 44–72%; I^2^ = 95.7%) across 16 studies (N = 1538 patients with confirmed diagnoses); six studies (Ji et al., 2019 [44]; Wu et al., 2023 [45]; Hakizimana et al., 2026 [46]; Abarca-Barriga et al., 2025 [47]; Monroe et al., 2016 [48]; Ilic et al., 2024 [49]) contributed to the yield analysis only.

### 3.1. Search Results

A total of 534 records were identified through database searches (PubMed, n = 335; Web of Science, n = 199), supplemented by 181 records identified through AI-assisted search, for a total of 715 records. After removing 214 duplicate records, 501 records were screened and 478 were excluded (population outside the NDD definition used in this review, technical variant data only, without post-diagnostic clinical outcomes, non-English-language publications, case reports with fewer than five patients, reviews or guidelines without primary data). The full list of excluded full-text studies, with individual reasons, is provided in Supplementary Table S6. Twenty-three studies were included from the original search, twenty-two of which entered quantitative meta-analysis. Pagan et al. (2017) [43] was retained for qualitative synthesis only, and six studies (Ji et al., 2019 [44]; Wu et al., 2023 [45]; Hakizimana et al., 2026 [46]; Abarca-Barriga et al., 2025 [47]; Monroe et al., 2016 [48]; Ilic et al., 2024 [49]) were included in the yield analysis but excluded from actionability (no formal actionability fraction reported). Despite inclusion of ADHD and language delay as search terms, no eligible study with ADHD or isolated developmental language delay as the primary cohort diagnosis was identified, reflecting a recognised gap in the evidence base. The selection process is summarised in Figure 1.

### 3.2. Quality Assessment

Quality was appraised with the JBI Critical Appraisal Checklist for Studies Reporting Prevalence Data (Table 1). Across the 22 studies entering quantitative synthesis and assessed with this instrument, the mean score was 7.18/9, with seventeen studies rated high quality, four (Xu et al., 2018 [63]; Henderson et al., 2014 [58]; Charouf et al., 2024 [42]; Ilic et al., 2024 [49]) rated moderate, and one (Saam et al., 2008 [57]) rated low. Strengths were near-universal in sample frame appropriateness (Q1), detailed description of subjects and setting (Q4), valid methods for condition identification (Q6), and appropriate statistical analysis (Q8), each satisfied by all 22 studies. The most consistent weakness was inadequate justification of sample size (Q3), unclear or unmet in every study; additional weaknesses clustered in data-analysis coverage (Q5) and response-rate handling (Q9), reflecting incomplete follow-up documentation (Henderson et al., 2014 [58]), non-consecutive, interview-based ascertainment (Saam et al., 2008 [57]), and an unexplained gap between the identified and tested cohort (Charouf et al., 2024 [42]). Pagan et al. (2017) was not assessed using the JBI Prevalence checklist, as its case–control biochemical design lacked a prespecified diagnostic-yield denominator required by this instrument; methodological reliability was instead considered narratively, and the study was retained for qualitative synthesis only (Section 2.3; Table 1) [43].

### 3.3. Characteristics of Included Studies

The studies were published between 2008 and 2026 and spanned China, Canada, Italy, the Republic of Korea, the USA, France, Israel, Rwanda, Lebanon, Peru, The Netherlands, and Slovenia/Spain (two-centre study). Designs included retrospective analyses and chart reviews, prospective cohort and multicentre studies, a physician-survey pilot, a semiautomated phenotype-driven WES workflow, and trio-based WES/WGS studies, including second-tier testing after negative CMA or standard WES. Sample sizes ranged from 17 (Monroe et al., 2016 [48]) to 1792 (Coulter et al., 2011 [64]). Most participants were children; Minardi et al. (2020) [41] remained the sole exclusively adult cohort (n = 71, DEE). Predominant diagnoses were ASD, ID, and GDD, frequently with comorbid congenital anomalies or epilepsy. Condensed study characteristics are presented in Table 2; the full study-by-study characteristics, the detailed summary of findings, and the complete summary of genotype-directed clinical actions are provided in Supplementary Materials (Tables S2 and S3).

### 3.4. Summary of Findings

#### 3.4.1. Diagnostic Yield—Primary Analysis

**Figure 2:**
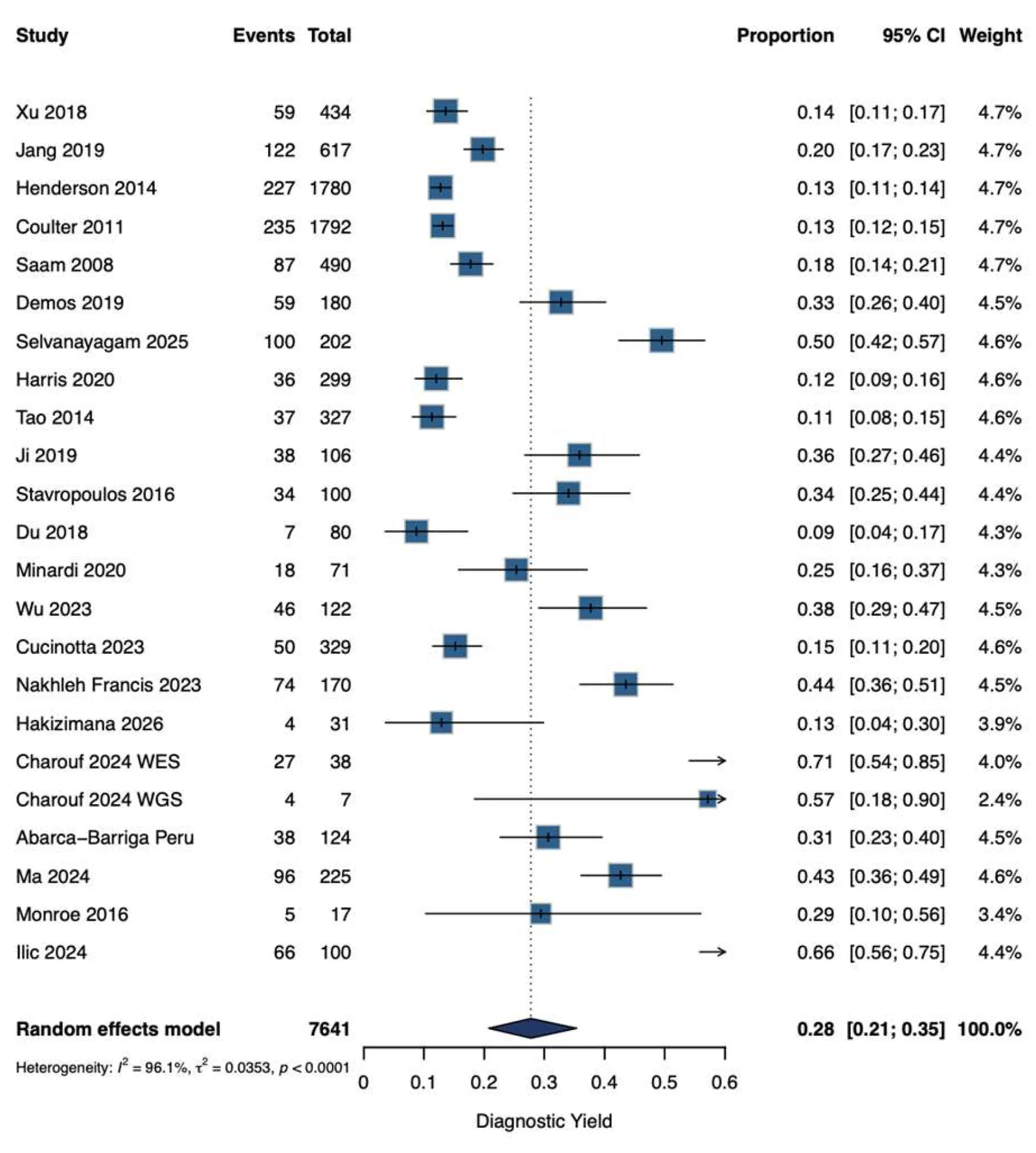
Forest plot of pooled diagnostic yield across 23 data points from 22 studies (N = 7641). Random-effects model, Freeman–Tukey transformation, REML (restricted maximum likelihood). Studies: Xu 2018 [63]; Jang 2019 [22]; Henderson 2014 [58]; Coulter 2011 [64]; Saam 2008 [57]; Demos 2019 [40]; Selvanayagam 2025 [17]; Harris 2020 [65]; Tao 2014 [66]; Ji 2019 [44]; Stavropoulos 2016 [67]; Du 2018 [68]; Minardi 2020 [41]; Wu 2023 [45]; Cucinotta 2023 [69]; Nakhleh Francis 2023 [56]; Hakizimana 2026 [46]; Charouf 2024, WES and WGS [42]; Abarca-Barriga 2025 [47]; Ma 2024 [70]; Monroe 2016 [48]; Ilic 2024 [49].

Pooled diagnostic yield across 23 data points from 22 studies (N = 7641) was 28% (95% CI 21–35%; I^2^ = 96.1%, Q *p* < 0.0001), reflecting substantial heterogeneity attributable to differences in modality, population composition, and variant classification. The highest individual yield (71.1%; Charouf et al., 2024 [42], WES arm) was observed in a highly selected cohort combining epilepsy and NDD phenotypes, consistent with the enrichment of pathogenic variants expected in such second-tier, phenotypically complex populations. Selvanayagam et al. (2025) [17] also reported a high yield (49.5%), reflecting a research return-of-results protocol not directly comparable to standard clinical yield. Restriction to methodologically homogeneous studies (flag = 1; n = 15) produced a similar estimate of 24% (95% CI 16–33%; I^2^ = 96.2%), indicating that the primary estimate is not substantially driven by non-standard definitions or ascertainment methods (Figure 2).

#### 3.4.2. Clinical Actionability—Primary Analysis

**Figure 3:**
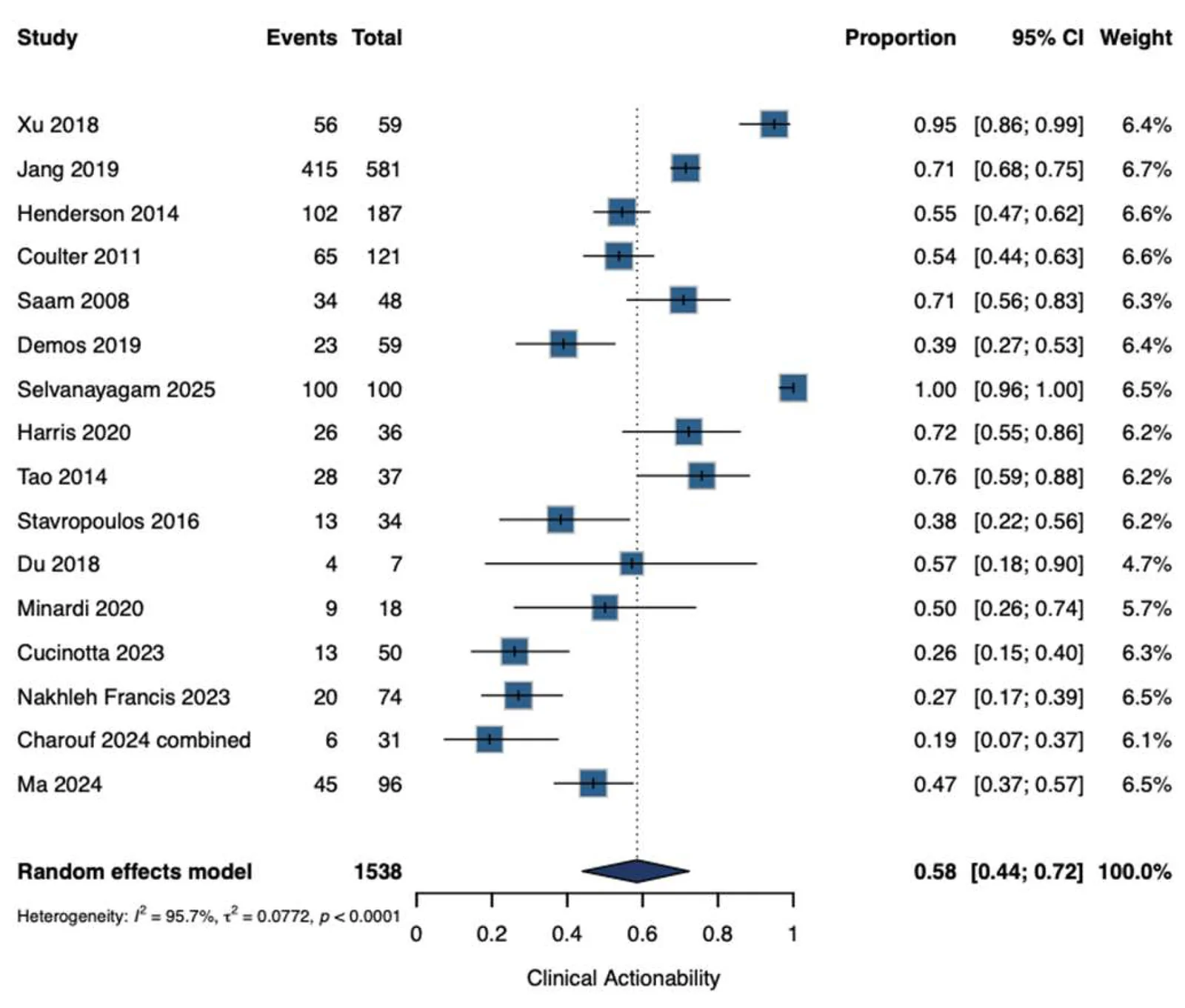
Forest plot of pooled clinical actionability across 16 studies (N = 1538 confirmed diagnoses). Studies: Xu 2018 [63]; Jang 2019 [22]; Henderson 2014 [58]; Coulter 2011 [64]; Saam 2008 [57]; Demos 2019 [40]; Selvanayagam 2025 [17]; Harris 2020 [65]; Tao 2014 [66]; Stavropoulos 2016 [67]; Du 2018 [68]; Minardi 2020 [41]; Cucinotta 2023 [69]; Nakhleh Francis 2023 [56]; Charouf 2024 (WES and WGS combined) [42]; Ma 2024 [70].

Pooled clinical actionability across 16 studies (N = 1538) was 58% (95% CI 44–72%; I^2^ = 95.7%, Q *p* < 0.0001). The estimate was reasonably robust across sensitivity analyses, ranging from 54% (95% CI 42–66%) when Selvanayagam et al. (2025) [17] was excluded to 64% (95% CI 48–78%) when epilepsy/DEE-associated cohorts (Demos et al., 2019 [40]; Minardi et al., 2020 [41]; Charouf et al., 2024 [42]) were excluded—a range of 10 percentage points. Restriction to methodologically homogeneous studies (flag = 1; n = 9) produced 59% (95% CI 43–74%), and exclusion of Nakhleh Francis et al. (2023) [56], whose actionability estimate reflected only the specialist-referral component of a non-aggregable multi-category framework, produced 61% (95% CI 46–75%); both changes were minimal, confirming that neither non-standard ascertainment nor this single non-comparable framework meaningfully drives the primary estimate (Figure 3). All sensitivity analyses are reported in Section 3.4.4.

#### 3.4.3. Subgroup Analysis by Testing Modality

**Figure 4:**
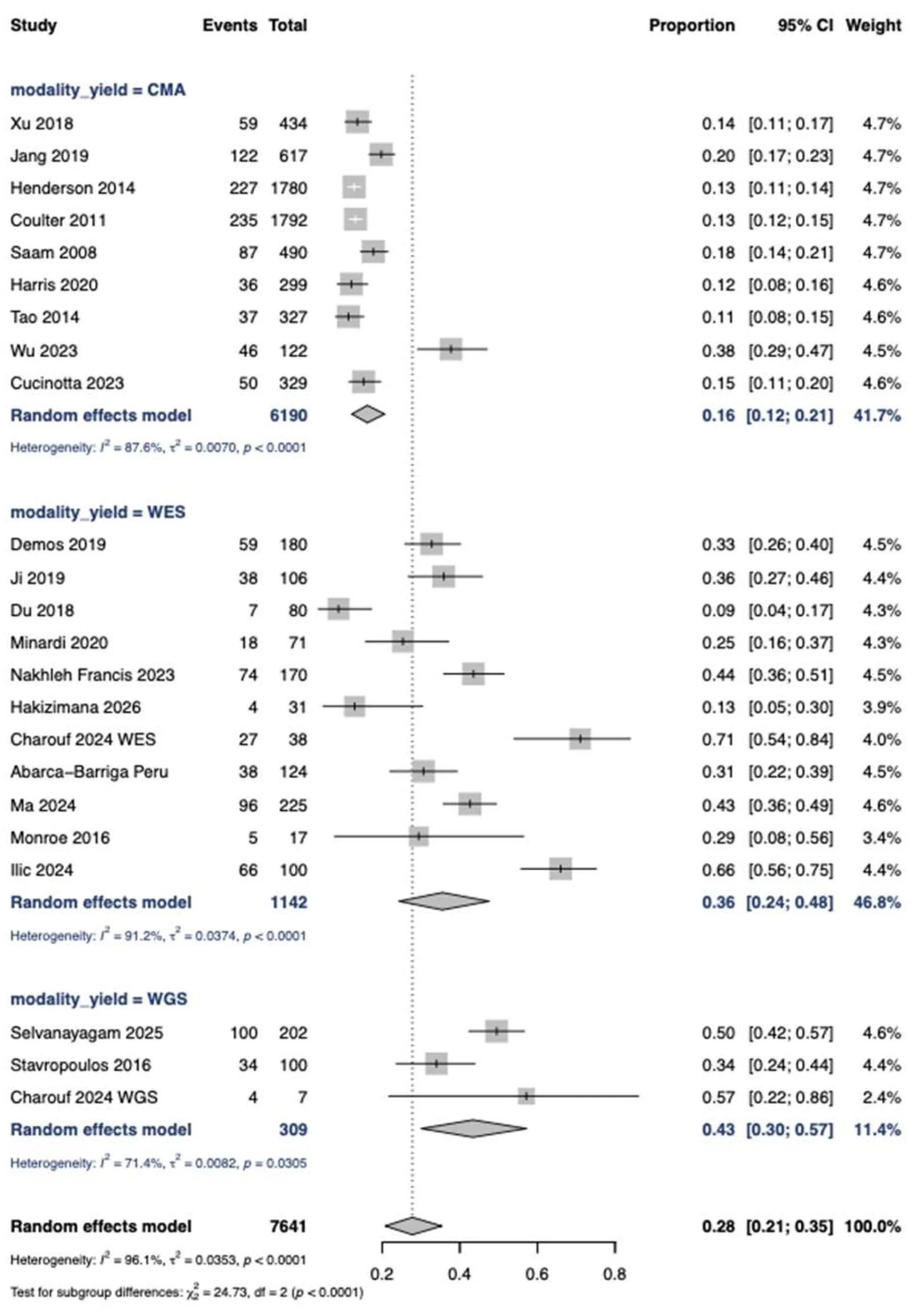
Subgroup forest plots of diagnostic yield by testing modality (23 data points from 22 studies). CMA, chromosomal microarray analysis; WES, whole exome sequencing; WGS, whole-genome sequencing. Studies: Xu 2018 [63]; Jang 2019 [22]; Henderson 2014 [58]; Coulter 2011 [64]; Saam 2008 [57]; Demos 2019 [40]; Selvanayagam 2025 [17]; Harris 2020 [65]; Tao 2014 [66]; Ji 2019 [44]; Stavropoulos 2016 [67]; Du 2018 [68]; Minardi 2020 [41]; Wu 2023 [45]; Cucinotta 2023 [69]; Nakhleh Francis 2023 [56]; Hakizimana 2026 [46]; Charouf 2024, WES and WGS [42]; Abarca-Barriga 2025 [47]; Ma 2024 [70]; Monroe 2016 [48]; Ilic 2024 [49].

A total of 7641 participants across 23 data points from 22 studies were included in the meta-analysis of diagnostic yield, stratified by testing modality. Nine studies (n = 6190) reported diagnostic yield using CMA, with a pooled proportion of 0.16 (95% CI: 0.12–0.21; I^2^ = 87.6%, *p* < 0.0001). Eleven studies (n = 1142) reported yield using WES, with a pooled proportion of 0.36 (95% CI: 0.24–0.48; I^2^ = 91.2%, *p* < 0.0001). Three studies (n = 309) reported yield using WGS, with a pooled proportion of 0.43 (95% CI: 0.30–0.57; I^2^ = 71.4%, *p* = 0.0305). Across all 23 data points, the overall pooled diagnostic yield was 0.28 (95% CI: 0.21–0.35; I^2^ = 96.1%, *p* < 0.0001), and a test for subgroup differences confirmed a statistically significant gradient across testing modalities (χ^2^ = 24.73, df = 2, *p* < 0.0001), with WGS and WES demonstrating numerically higher diagnostic yields than CMA (Figure 4). This yield gradient should not, however, be read as a direct indicator of clinical value: as detailed below, the corresponding actionability gradient runs in the opposite direction for WES, with CMA showing numerically higher actionability than WES despite its lower diagnostic yield—underscoring that a higher rate of genetic diagnosis does not automatically translate into a higher rate of actionable clinical findings.

A total of 1538 participants across 16 studies were included in the meta-analysis of clinical actionability, stratified by testing modality (CMA, WES, and WGS); one study (Charouf et al., 2024) [42] combined WES and WGS testing and could not be allocated to a single modality subgroup and was therefore excluded from this stratified analysis while remaining in the primary pooled estimate. Eight studies (n = 1119) reported clinical actionability using CMA, including Xu et al. (2018) [63], Jang et al. (2019) [22], Henderson et al. (2014) [58], Coulter et al. (2011) [64], Saam et al. (2008) [57], Harris et al. (2020) [65], Tao et al. (2014) [66], and Cucinotta et al. (2023) [69]. The pooled proportion of positive findings was 0.66 (95% CI: 0.51–0.80), with substantial heterogeneity across studies (I^2^ = 92.7%, τ^2^ = 0.0464, *p* < 0.0001). Five studies (n = 254) reported clinical actionability using WES: Demos et al. (2019) [40], Du et al. (2018) [68], Minardi et al. (2020) [41], Nakhleh Francis et al. (2023) [56], and Ma et al. (2024) [70]. The pooled proportion was 0.40 (95% CI: 0.30–0.51), with moderate heterogeneity (I^2^ = 54.8%, τ^2^ = 0.0066, *p* = 0.0652). Two studies (n = 134) reported clinical actionability using WGS: Selvanayagam et al. (2025) [17] and Stavropoulos et al. (2016) [67]. The pooled proportion was 0.80 (95% CI: 0.06–1.00), reflecting substantial between-study heterogeneity (I^2^ = 98.7%, τ^2^ = 0.3572, *p* < 0.0001) and a very wide confidence interval, largely driven by the discrepancy between the two included studies (1.00 [0.96–1.00] vs. 0.38 [0.22–0.56]).

**Figure 5:**
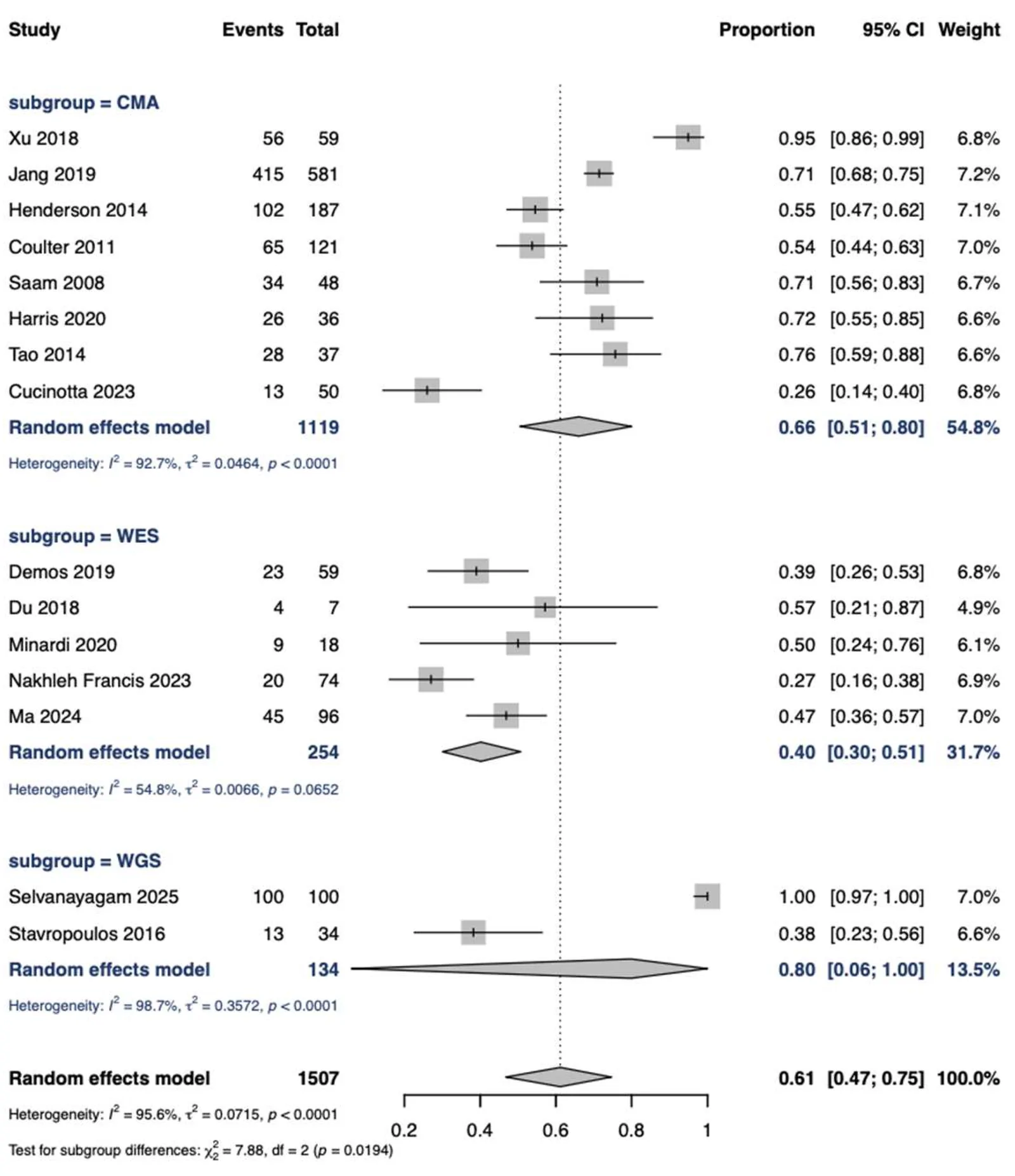
Subgroup forest plots of clinical actionability by genetic testing modality (15 studies; one study with combined WES/WGS testing excluded from subgroup allocation—see Section 3.4.3). Squares indicate study-specific proportions and horizontal lines their 95% confidence intervals; diamonds indicate pooled estimates for each modality subgroup and for all studies combined. Studies: Xu 2018 [63]; Jang 2019 [22]; Henderson 2014 [58]; Coulter 2011 [64]; Saam 2008 [57]; Harris 2020 [65]; Tao 2014 [66]; Cucinotta 2023 [69]; Demos 2019 [40]; Du 2018 [68]; Minardi 2020 [41]; Nakhleh Francis 2023 [56]; Ma 2024 [70]; Selvanayagam 2025 [17]; Stavropoulos 2016 [67].

Across the 15 studies included in the subgroup analysis (N = 1507), the overall pooled clinical actionability using a random-effects model was 0.61 (95% CI: 0.47–0.75), with substantial heterogeneity (I^2^ = 95.6%, τ^2^ = 0.0715, *p* < 0.0001). A test for subgroup differences indicated a statistically significant difference in clinical actionability across the three testing modalities (χ^2^ = 7.88, df = 2, *p* = 0.0194), with CMA and WGS demonstrating numerically higher levels of clinical actionability than WES, as shown in Figure 5.

#### 3.4.4. Sensitivity Analyses

Results of all subgroup and sensitivity analyses are presented in Table 3. Restricting the diagnostic-yield analysis to the fifteen methodologically homogeneous studies (flag = 1) produced a similar pooled estimate of 24% (95% CI: 16–33%; I^2^ = 96.2%, *p* < 0.0001), indicating that the primary yield estimate is not substantially driven by methodologically heterogeneous studies. Exclusion of the three epilepsy/DEE-associated cohorts (Demos et al., 2019 [40]; Minardi et al., 2020 [41]; Charouf et al., 2024 [42]) similarly had minimal impact, yielding 25% (95% CI: 18–33%; I^2^ = 96.4%, *p* < 0.0001).

Restricting the clinical-actionability analysis to the nine methodologically homogeneous studies (flag = 1) produced a similar pooled estimate of 59% (95% CI: 43–74%; I^2^ = 91.3%, *p* < 0.0001), supporting the robustness of the primary actionability estimate. Given that Selvanayagam et al. (2025) [17] employed a broader three-category actionability framework, a sensitivity analysis excluding this study yielded a modestly lower pooled estimate of 54% (95% CI: 42–66%; I^2^ = 92.9%, *p* < 0.0001), indicating that this study contributed approximately 4 percentage points to the primary pooled estimate. Exclusion of the three epilepsy/DEE-associated cohorts resulted in a higher pooled estimate of 64% (95% CI: 48–78%; I^2^ = 96.0%, *p* < 0.0001), consistent with these cohorts exerting a downward influence on the primary estimate. Because Nakhleh Francis et al. (2023) [56] reported actionability using only the specialist-referral component of a non-aggregable, four-category framework, a further sensitivity analysis excluding this study was performed, yielding 61% (95% CI: 46–75%; I^2^ = 95.4%, *p* < 0.0001)—a minimal change confirming that this non-standard ascertainment does not meaningfully drive the primary estimate.

The subgroup Q-between test for clinical actionability was statistically significant (χ^2^ = 7.88, df = 2, *p* = 0.0194; Section 3.4.3, Figure 5), confirming a dissociation between diagnostic yield and clinical actionability by modality: despite CMA demonstrating the lowest diagnostic yield among the three modalities, it showed numerically higher clinical actionability than WES. This finding suggests that a positive result obtained via CMA is more likely to be clinically actionable than a positive result obtained via WES, even though WES identifies a higher overall proportion of positive findings; the WGS actionability estimate, based on only two studies with markedly different frameworks, remains too imprecise to interpret with confidence.

To assess robustness to the choice of variance-stabilising transformation, sensitivity analyses using logit-transformed proportions and a generalised linear mixed model (GLMM) were performed for both outcomes. Pooled estimates were consistent across all three approaches for diagnostic yield (Freeman–Tukey: 28%; logit: 26.8%; GLMM: 26.6%) and clinical actionability (Freeman–Tukey: 58%; logit: 57.7%; GLMM: 61.1%), indicating that the primary findings are not driven by the Freeman–Tukey transformation (Supplementary Figures S1 and S2, Table S1). Given the high heterogeneity observed for both outcomes, 95% prediction intervals were additionally calculated to estimate the range of true effects expected in a new clinical setting. These intervals were substantially wider than the corresponding confidence intervals for both diagnostic yield (2–67% vs. 21–35%) and clinical actionability (5–100% vs. 44–72%), underscoring that the pooled point estimates should not be extrapolated to any single new population without considering this much broader range of plausible values (Supplementary Figures S3 and S4).

The full annotated version, including per-analysis notes, is provided in Supplementary Table S1.

#### 3.4.5. Publication Bias

**Figure 6:**
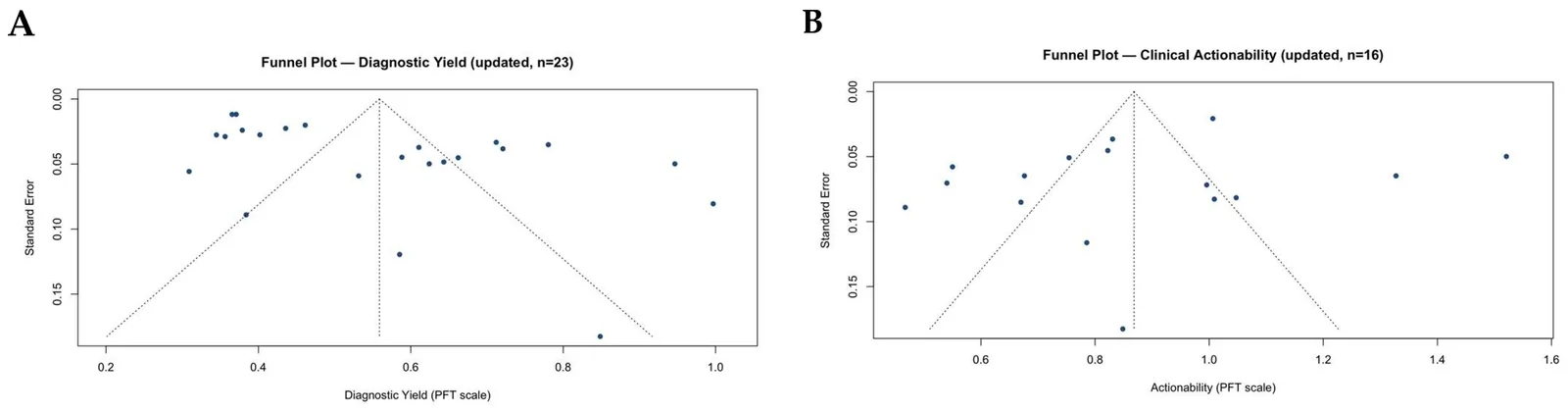
Funnel plots. (**A**) Diagnostic yield (23 data points from 22 studies). (**B**) Clinical actionability (16 studies).

Egger’s test indicated significant funnel-plot asymmetry for diagnostic yield (*p* = 0.0008) but not for clinical actionability (*p* = 0.3307). For yield, one plausible explanation is structural heterogeneity—WGS and WES studies with inherently higher yields were also smaller in sample size—though the source of funnel-plot asymmetry cannot be confidently determined from the available data (Figure 6A). The number of studies available (k = 23 for yield, k = 16 for actionability) supports reasonably interpretable bias-detection tests, though results should still be interpreted with appropriate caution given the number of studies involved. No comparable asymmetry was observed for clinical actionability (Figure 6B).

### 3.5. Certainty of Evidence

Using the GRADE approach adapted for single-group prevalence/proportion meta-analyses in the absence of a comparator group, the overall certainty of evidence was rated very low for both diagnostic yield and clinical actionability, reflecting downgrades for risk of bias, very serious inconsistency (I^2^ > 95% for both outcomes), indirectness (non-standard outcome definitions in a subset of studies), and imprecision (wide 95% confidence intervals). Full domain-level ratings are provided in Supplementary Table S4.

## 4. Discussion

In this systematic review and meta-analysis, we assembled evidence on how genetic testing informs clinical and therapeutic management—diagnosis, intervention, and monitoring—in patients with NDDs, with a primary focus on ASD, ID, and GDD; the included studies document management changes but rarely long-term outcome improvement. The following sections reflect on diagnostic yield, actionability, the diagnostic odyssey, and barriers to implementation.

### 4.1. Beyond the Numbers of the Diagnostic Yield

The pooled diagnostic yield of 28% (95% CI 21–35%; I^2^ = 96.1%) is consistent with large-scale NDD meta-analyses, where CMA yields 15–20% and NGS-based methods achieve higher yields [5,37,71]. The CMA subgroup yield of 16% (95% CI 12–21%) falls within the historical 15–20% range [21], reflecting microarray’s limited sensitivity for single-nucleotide variants. The WES subgroup yield of 36% (95% CI 24–48%) sits at the lower bound of the 36–53% benchmark for first-tier exome sequencing [23], which is plausible given that several included WES cohorts were epilepsy-specific or second-tier populations rather than unselected NDD referrals; the highest individual yield in the dataset (71.1%; Charouf et al., 2024 [42]) likewise arose in such a highly selected epilepsy-plus-NDD cohort. The WGS yield of 43% (95% CI 30–57%; k = 3) was numerically highest, consistent with the CMA→WES→WGS gradient [5,23,37]. The gradient was statistically significant (Q-between χ^2^ = 24.73, *p* < 0.0001), extending prior findings beyond predominantly GDD/ID populations. It should nonetheless be interpreted cautiously: modality is confounded with testing era, referral criteria, phenotypic severity, cohort selection, and position in the diagnostic pathway (first-line versus after prior negative testing), and the WGS estimate rests on only three studies. Moreover, the 95% prediction interval (2–67%) shows that the yield expected in any single new setting is far more variable than the confidence interval of the pooled mean suggests, so the pooled estimate should not be read as a point expectation for an individual clinical service. Importantly, a “negative” result should not be equated with the absence of genetic causation; reanalysis can substantially increase yield as gene–disease knowledge evolves [72].

### 4.2. The Shift from Diagnostic Labelling to Actionable Management

Clinical actions prompted by genetic diagnoses followed five strategic directions: pharmacological adjustment (e.g., targeted rather than empirical medication [73], avoidance of contraindicated agents such as sodium-channel blockers in *SCN1A* [42,74], and targeted use of carbamazepine in *KCNQ2*-related neonatal epilepsy [75]); dietary and metabolic intervention (e.g., melatonin for *ASMT* deficiency in qualitative synthesis [43], or a pyridoxine and lysine-restricted diet for *ALDH7A1* [40]); comorbidity management (e.g., cardiology referral in *KCNQ1* variants [44], thyroxine replacement in 1p36 deletion [66]); reproductive and family-focused actions (e.g., 22 reproductive-decision changes among the 96 diagnosed patients in Ma et al. [70]); and clinical efficiency (avoiding redundant invasive tests and ending the diagnostic odyssey through molecular confirmation). These examples derive from specific genes and syndromes and should not be taken to imply that comparable targeted therapy exists for most molecular diagnoses. Throughout this Discussion section, “clinical actionability” denotes the proportion of diagnosed patients with at least one documented management action attributed to the genetic finding (Section 2.4), whereas “clinical utility” is reserved for the broader, outcome-based concept that the included studies did not measure. The pooled actionability of 58% (95% CI 44–72%) indicates that for roughly three of five patients with a confirmed diagnosis, at least one management action was reported as attributable to the genetic finding, and this was stable across sensitivity analyses (54–64%). This figure describes the frequency of documented actions, not the magnitude of their benefit, and the 95% prediction interval (5–100%) indicates very wide variation between clinical settings.

A notable dissociation emerged between yield and actionability by modality. CMA showed the highest interpretable actionability (66%, 95% CI 51–80%) despite the lowest yield (16%), plausibly reflecting mature clinical protocols and established management guidelines for copy number variant (CNV)-associated syndromes. WES actionability (40%, 95% CI 30–51%) was lower, which may reflect the epilepsy-specific and second-tier composition of the WES cohorts and also definitional differences: Nakhleh Francis et al. [56], for example, contributed only the specialist-referral component (27%) of a multi-category outcome, and actions were not consistently stratified by type (referral or further investigation, surveillance, or disease-specific therapy) across studies. The WGS actionability estimate (80%, 95% CI 6–100%) was uninterpretable, driven by markedly different definitions in the two included studies. Because actionability definitions, ascertainment methods, and testing position differed between studies, the CMA-versus-WES comparison should be considered hypothesis-generating rather than evidence that one modality yields more clinically useful results than the other. These findings suggest that diagnostic-classification quality and the maturity of downstream management pathways, not yield alone, may shape downstream action.

### 4.3. Ending the Diagnostic Odyssey: Psychological and Economic Impact

The diagnostic odyssey—the protracted path across specialties and disconnected evaluations—may end when molecular diagnosis clarifies prognosis and eliminates needless testing, reducing cumulative costs [48,76,77]. Concrete examples include cessation of recurrent endocrine testing after molecular confirmation of Sotos syndrome (*NSD1*) and the resolution of a 38.7-year median diagnostic odyssey in adults with DEE (Minardi et al., 2020 [41]). Few included studies quantified economic effects: in a small pilot in intellectual disability, trio-WES cost $3972 per patient compared with $16,409 for the traditional diagnostic trajectory, with average savings of $3547 in genetic and metabolic investigations among diagnosed patients (Monroe et al. [48]); in early-onset epilepsy, time to diagnosis was shorter when WES was performed early (mean 145 days in the prospective versus 2882 days in the retrospective group), with potential savings of CAD 1290–5110 per patient (Demos et al. [40]). Beyond economics, a confirmed diagnosis is reported to enhance parental empowerment and enable informed counselling [78,79], although none of the included studies measured psychological outcomes directly (Section 4.5); reproductive counselling, an often-overlooked actionable objective, can substantially alter recurrence-risk estimates and 23% of diagnosed patients in Nakhleh Francis et al. [56]. The value of testing thus lies in transforming uncertainty into concrete family-management strategies, although its economic and psychological benefits remain largely inferred rather than measured.

### 4.4. Barriers to Implementation and the VUS Dilemma

Implementation remains uneven, with disparities by comorbidity, region, and sociodemographic factors and low rates of guideline-concordant testing [80,81,82,83,84]. A central barrier is the VUS dilemma: ambiguous findings generate anxiety and infrequent management change for some variants, and their handling shapes reported yield (12.9% versus 29.0% when phenotype-concordant VUS were counted in Hakizimana et al. [46]; VUS were 1.8 times as frequent as pathogenic or likely pathogenic CNVs in Cucinotta et al. [69]). Management change after uncertain results should not, however, be read as clinical benefit. In Jang et al. (2019) [22], management changes were recorded for 86% of patients with variants of possible significance, but also for 67.3% of those with benign variants, indicating that these figures largely reflect general clinical activity after any microarray result; in Coulter et al. [64] and Tao et al. [66], by contrast, uncertain findings prompted far fewer actions than pathogenic ones (34% versus 54%, and one patient, respectively). Increased surveillance or referral after a VUS may reflect clinical caution rather than genomic actionability, and greater medical activity is not demonstrated benefit. Because Jang et al. pooled all variant classes, it was excluded from the flag = 1 sensitivity analysis, in which the pooled actionability estimate was essentially unchanged (59%). Standardised ACMG/Association for Molecular Pathology (AMP) frameworks formalise why many variants remain VUS pending evidence, reinforcing the need for careful counselling, segregation studies, and planned reinterpretation [85].

### 4.5. Limits of Quantifying Clinical Utility Beyond Yield and Actionability

Despite frequent emphasis on the utility of genetic testing, objective quantitative data beyond yield and actionability remain scarce [33,86,87,88,89,90]. While ES/genome sequencing (GS)/WGS can end the diagnostic journey (with management modified in 39% of early-onset epilepsy and 50% of adult DEE cases), such studies, much like earlier CMA-based paediatric cohorts reporting comparable management-change rates, rarely report standardised data on quality of life, long-term outcomes, or comprehensive economic benefit [19,40,41,66,87,88]. Among the newly included studies, outcome-related information was limited to costs (Section 4.3), the yield of additionally requested investigations (12 of 25 positive in Cucinotta et al. [69]), and descriptive reports of improved seizure control and neurodevelopmental trajectories in six children (Charouf et al. [42]). Clinical actionability in this review is therefore a proxy for, not a measure of, clinical utility: an action being taken does not demonstrate that outcomes improved. The “meaningful” value of genomic tools is often conveyed through illustrative cases rather than longitudinal metrics [17,79,91,92], underscoring the need for prospective research linking genetically guided management to measurable long-term outcomes [41,58,87,88,93].

A further, largely unmeasured layer is post-result phenotypic reassessment. Reverse phenotyping—re-examining the patient’s clinical features in light of a candidate genomic finding—can turn an ambiguous variant into a diagnosis or reveal subtle, atypical, or age-dependent features and may therefore influence both yield and downstream management. Of the 13 sequencing studies included, only Ilic et al. [49] described it as a formal step: reverse phenotyping was indicated in 33 of 100 children after WES (half of those with positive findings), and the need for it was significantly higher in children with previously negative genetic testing (*p* = 0.013), although WES yield itself did not differ by prior testing (*p* = 0.381). None of the other twelve sequencing studies described reverse phenotyping, and how often a molecular diagnosis was clinically unexpected was not reported systematically. Differences in post-sequencing reassessment may therefore contribute to the heterogeneity in reported yield, and this could not be assessed here.

### 4.6. Limitations

Several limitations warrant acknowledgement. First, substantial heterogeneity was observed for both outcomes (I^2^ = 96.1% for yield; 95.7% for actionability), reflecting genuine differences in cohort composition, modality, variant-classification standards, and diagnostic era, and addressed through the random-effects model and sensitivity analyses. Second, no included study contained an untreated control group, precluding comparative effect-size estimation; the PICO comparison was addressed indirectly through reported management-change rates, consistent with prior genomic-medicine meta-analyses [37,94]. Third, clinical actionability was operationalised heterogeneously—non-standard denominators (Jang et al., 2019 [22]; Saam et al., 2008 [57]; Henderson et al., 2014 [58]; Nakhleh Francis et al., 2023 [56]) and the broad three-category framework of Selvanayagam et al. (2025) [17]—and addressed through flag = 1 sensitivity analysis, although actions could not be consistently stratified by type (referral or further investigation, surveillance, or disease-specific therapy); for example, Charouf et al. (2024) [42] counted only aetiology-specific treatment changes, whereas Nakhleh Francis et al. (2023) [56] counted only specialist referrals. Fourth, six studies (Ji et al., 2019 [44]; Wu et al., 2023 [45]; Hakizimana et al., 2026 [46]; Abarca-Barriga et al., 2025 [47]; Monroe et al., 2016 [48]; Ilic et al., 2024 [49]) did not report a formal actionability fraction. Fifth, Egger’s test indicated yield asymmetry (*p* = 0.0008), likely structural rather than selective, though publication bias cannot be excluded; the number of studies available (k = 23/16) improves but does not eliminate this limitation. Sixth, testing position within the diagnostic pathway (first-line vs. second-tier, i.e., following prior negative genetic testing) could not be systematically ascertained across all included studies; where reported, testing position varied (e.g., Charouf et al., 2024 [42], first-line NGS; Nakhleh Francis et al., 2023 [56], second-tier ES; a subset of patients in Demos et al., 2019 [40], and Abarca-Barriga et al., 2025 [47], had undergone prior negative testing), and this heterogeneity may contribute to the observed variability in diagnostic yield; similarly, only one included study (Ilic et al., 2024 [49]) reported reverse phenotyping, so differences in post-sequencing phenotypic reassessment could not be evaluated as a source of heterogeneity. Seventh, the included studies span 2008–2026, a period in which sequencing technologies, variant-classification standards, and clinical practice changed substantially, and they come from health systems that differ in access to specialists, treatments, and follow-up; temporal and geographical heterogeneity may therefore contribute materially to the pooled estimates and could not be evaluated formally. Eighth, the search was restricted to English-language publications and to PubMed and Web of Science supplemented by an AI-assisted search, so relevant studies indexed elsewhere (e.g., Embase or Scopus) may have been missed; moreover, yield and actionability were estimated from partly different sets of studies (23 and 16, respectively), so the two estimates do not describe identical populations, and overlap of patients between reports from the same institutions could not be excluded. Finally, checklist-based quality assessment is subject to inter-rater variability.

## 5. Conclusions

For patients with NDDs, primarily ASD, ID, and GDD, genetic testing has evolved from a tool for etiological labelling into an increasingly important component of personalised clinical and therapeutic management, although the certainty of this evidence is very low according to GRADE, reflecting substantial heterogeneity and study-level limitations.

Pooled diagnostic yield was 28% (95% CI 21–35%), with a significant gradient across modalities (CMA 16%, WES 36%, WGS 43%; *p* < 0.0001), and at least one management action was documented in 58% (95% CI 44–72%) of patients with a confirmed diagnosis, a figure stable across sensitivity analyses (54–64%) but accompanied by wide prediction intervals (yield 2–67%; actionability 5–100%) that limit extrapolation to individual settings. Personalisation manifested through optimised pharmacological selection, targeted metabolic intervention, proactive surveillance for systemic comorbidities, and reproductive counselling, while ending the diagnostic odyssey and providing prognostic clarity. Because clinical actionability is a proxy for, rather than a measure of, improved outcomes, these findings show that molecular diagnosis frequently prompts changes in management, not that it improves patient outcomes. Despite barriers—the VUS dilemma, cost, and bioinformatic demands—molecular diagnosis is an important step toward precision medicine in NDDs. Future priorities include (1) prospective studies incorporating untested control groups; (2) longitudinal assessment of outcomes following genetically guided management change; (3) a consensus operational definition of clinical actionability in neurodevelopmental genomics; and (4) reporting of testing position in the diagnostic pathway and of post-result phenotypic reassessment.

## Figures and Tables

**Table 1:** Quality assessment using the JBI Critical Appraisal Checklist for studies reporting prevalence data.

Study	Q1	Q2	Q3	Q4	Q5	Q6	Q7	Q8	Q9	Score	Quality
Xu 2018 [63]	Y	Y	U	Y	U	Y	Y	Y	U	6/9	Moderate
Jang 2019 [22]	Y	Y	U	Y	Y	Y	Y	Y	Y	8/9	High
Henderson 2014 [58]	Y	Y	U	Y	N	Y	U	Y	N	5/9	Moderate
Coulter 2011 [64]	Y	Y	U	Y	Y	Y	Y	Y	Y	8/9	High
Saam 2008 [57]	Y	N	N	U	N	Y	N	Y	N	3/9	Low
Demos 2019 [40]	Y	Y	U	Y	Y	Y	Y	Y	Y	8/9	High
Selvanayagam 2025 [17]	Y	Y	U	Y	Y	Y	Y	Y	Y	8/9	High
Harris 2020 [65]	Y	Y	U	Y	N	Y	Y	Y	Y	7/9	High
Tao 2014 [66]	Y	Y	U	Y	Y	Y	Y	Y	Y	8/9	High
Ji 2019 [44]	Y	Y	U	Y	Y	Y	Y	Y	Y	8/9	High
Stavropoulos 2016 [67]	Y	Y	U	Y	Y	Y	Y	Y	Y	8/9	High
Du 2018 [68]	Y	Y	N	Y	Y	Y	Y	U	Y	7/9	High
Minardi 2020 [41]	Y	Y	U	Y	Y	Y	Y	Y	Y	8/9	High
Wu et al., 2023 [45]	Y	Y	U	Y	Y	Y	Y	Y	Y	8/9	High
Cucinotta et al., 2023 [69]	Y	Y	U	Y	Y	Y	Y	Y	Y	8/9	High
Nakhleh Francis et al., 2023 [56]	Y	Y	U	Y	Y	Y	Y	Y	Y	8/9	High
Hakizimana et al., 2026 [46]	Y	U	U	Y	Y	Y	Y	Y	Y	7/9	High
Charouf et al., 2024 [42]	Y	U	U	Y	Y	Y	Y	Y	U	6/9	Moderate
Abarca-Barriga et al., 2025 [47]	Y	U	U	Y	Y	Y	Y	Y	Y	7/9	High
Ma et al., 2024 [70]	Y	Y	U	Y	Y	Y	Y	Y	Y	8/9	High
Monroe et al., 2016 [48]	Y	Y	N	Y	Y	Y	Y	Y	Y	8/9	High
Ilic et al., 2024 [49]	Y	U	U	Y	Y	Y	Y	Y	Y	6/9	Moderate
Pagan et al., 2017 [43]	-	-	-	-	-	-	-	-	-	N/A *	

JBI, Joanna Briggs Institute Critical Appraisal Checklist for Studies Reporting Prevalence Data. Q1: Was the sample frame appropriate to address the target population? Q2: Were study participants sampled in an appropriate way? Q3: Was the sample size adequate? Q4: Were the study subjects and the setting described in detail? Q5: Was the data analysis conducted with sufficient coverage of the identified sample? Q6: Were valid methods used for the identification of the condition? Q7: Was the condition measured in a standard, reliable way for all participants? Q8: Was there appropriate statistical analysis? Q9: Was the response rate adequate, and if not, was the low response rate managed appropriately? Y = Yes; N = No; U = Unclear. Studies scoring ≥7 = High; 5–6 = Moderate; <5 = Low. * Pagan et al. 2017 [43] was not assessed using the JBI Prevalence checklist, which requires a quantifiable prevalence/proportion denominator absent in this study (no significant yield could be calculated, *p* = 0.56 vs. controls). Methodological reliability was instead considered narratively: the study used a case–control biochemical design without a pre-specified diagnostic-yield denominator, limiting comparability to the prevalence-focused studies in this review; it is retained for qualitative synthesis only (Section 2.3). Overall certainty of evidence, assessed using GRADE (Supplementary Table S4), was very low for both diagnostic yield and clinical actionability, reflecting observational study design, serious risk of bias, and substantial unexplained heterogeneity.

**Table 2:** Condensed characteristics of the included studies.

Study	Design	Population (N, Diagnosis)	Method	Yield (%)	Action. n/N (%)	Ascertainment ‖
Xu 2018 [63]	Retrospective	434; DD/ID & ASD	CMA	13.6	56/59 (94.9)	Retrospective
Jang 2019 [22]	Prospective multicentre	617; DD/ID, ASD, MCA	CMA	19.8	415/581 (71.4)	Prospective
Henderson 2014 [58]	Retrospective (EMR)	1780; MCA, DD/ID, ASD	CMA	12.7	102/187 (54.5)	Retrospective
Coulter 2011 [64]	Retrospective chart	1792; DD/ID, ASD, anomalies	CMA	13.1	65/121 (54)	Retrospective
Saam 2008 [57]	Pilot/survey	48; DD/ID	aCGH	17.6	34/48 (70.8)	Retrospective (interview)
Demos 2019 [40]	Cohort, classified by epilepsy duration at WES	180; early-onset epilepsy	WES	33	23/59 (39)	Not stated
Selvanayagam 2025 [17]	Research cohort; retrospective review of returned results	202; ASD	WGS	49.5 *	100/100 (100) †	Retrospective
Harris 2020 [65]	Retrospective chart	500; ASD toddlers	CMA + FraX	12.0	26/36 (72.2)	Retrospective
Tao 2014 [66]	Retrospective medical-record review	327; ID/DD, ASD, MCA	CMA	11.3	28/37 (75.7)	Retrospective
Ji 2019 [44]	Phenotype-driven WES	106; suspected genetic	WES	36	-‡	N/A
Stavropoulos 2016 [67]	Prospective cohort	100; referred for genetics	WGS	34	13/34 (38)	Prospective
Du 2018 [68]	Trio WES post-CMA	80; ASD	WES	8.8	4/7 (57)	Not stated
Minardi 2020 [41]	Cohort (adults with DEE)	71 adults; DEE	WES	25.3	9/18 (50)	Not stated
Wu et al., 2023 [45]	Retrospective cohort	122; DD/ID moderate–severe	CMA	37.7	-‡	N/A
Cucinotta et al., 2023 [69]	Cohort (recruited at a tertiary service)	329; ASD	CMA (aCGH)	15.2	13/50 (26.0)	Not stated
Nakhleh Francis et al., 2023 [56]	Retrospective cohort	170; GDD	Trio-ES	43.5	20/74 (27.0) §	Retrospective
Hakizimana et al., 2026 [46]	Retrospective cohort	31; ASD (+ID 96.8%)	Trio-WES	12.9	-‡	N/A
Charouf et al., 2024 (WES) [42]	Retrospective cohort	38; Epilepsy + NDD	WES	71.1	6/31 (19.4) ¶	Retrospective
Charouf et al., 2024 (WGS) [42]	Retrospective cohort	7; Epilepsy + NDD	WGS	57.1	6/31 (19.4) ¶	Retrospective
Abarca-Barriga et al., 2025 [47]	Cross-sectional (descriptive)	124; ID	WES	30.6	-‡	N/A
Ma et al., 2024 [70]	Retrospective cohort	225; DD/ID	WES	42.7	45/96 (46.9)	Retrospective
Monroe et al., 2016 [48]	Pilot cohort (random selection)	17; ID	Trio-WES	29.4	-‡	N/A
Ilic et al., 2024 [49]	Retrospective cohort	100; DD/ID	WES	66.0	-**	N/A

* Yield reflects research return-of-results protocol. † For Selvanayagam et al. (2025) [17], actionability reflects a broad three-category framework (not directly comparable to the standard definition used elsewhere); denominator = 100/100 by protocol design. ‡ No formal actionability fraction reported; included in yield analysis only. § Actionability reflects specialist-referral component only (non-aggregable multi-category framework); see Section 2.3 and Section 2.4. ¶ Aetiology-specific treatment changes (medication or dietary) in 6 of 31 diagnosed children, combined across WES and WGS arms (not separable by modality). Specialist referrals (15 patients) are not included; see Section 2.3. ** Not formally reported; reverse phenotyping data (33/100) discussed narratively in Section 4. Full characteristics in Supplementary Table S2. ASD = autism spectrum disorder; DD/ID = Developmental Delay/intellectual disability; DEE = Developmental and Epileptic Encephalopathy; CMA = Chromosomal Microarray; WES = Whole-Exome Sequencing; WGS = Whole-Genome Sequencing; GDD = global developmental delay; DD = developmental delay; ID = intellectual disability; MCA = multiple congenital anomalies; aCGH = array comparative genomic hybridization; FraX = fragile X syndrome; ES = exome sequencing; EMR = electronic medical record. ‖ Ascertainment reflects how clinical actionability specifically was captured (chart/record review = Retrospective; contemporaneous clinical or research protocol = Prospective), which may differ from the overall study design listed in the Design column (e.g., Saam et al. 2008 [57], where actionability was ascertained through physician interviews after results had been issued); “Not stated” indicates that the source article does not specify how management changes were ascertained.

**Table 3:** Meta-analytic subgroup and sensitivity analyses.

Analysis	n	Estimate	95% CI	I^2^	*p*
**Primary analyses**					
Diagnostic yield—primary	23	28%	21–35%	96.1%	<0.0001
Clinical actionability—primary	16	58%	44–72%	95.7%	<0.0001
**Subgroup analyses—Diagnostic Yield (** Section 3.4.3 **)**					
Yield—CMA subgroup	9	16%	12–21%	87.6%	<0.0001
Yield—WES subgroup	11	36%	24–48%	91.2%	<0.0001
Yield—WGS subgroup	3	43%	30–57%	71.4%	0.0305
Yield—subgroup differences (χ^2^ = 24.73, df = 2)	-	-	-	-	<0.0001
**Subgroup analyses—Clinical Actionability (** Section 3.4.3 **)**					
Actionability—CMA subgroup	8	66%	51–80%	92.7%	<0.0001
Actionability—WES subgroup	5	40%	30–51%	54.8%	0.0652
Actionability—WGS subgroup	2	80%	6–100%	98.7%	<0.0001
Actionability—subgroup differences (χ^2^ = 7.88, df = 2)	-	-	-	-	0.0194
**Sensitivity analyses (** Section 3.4.4 **)**					
Yield—flag = 1 (methodologically homogeneous)	15	24%	16–33%	96.2%	<0.0001
Actionability—flag = 1 (methodologically homogeneous)	9	59%	43–74%	91.3%	<0.0001
Actionability—excl. Selvanayagam 2025 [17]	15	54%	42–66%	92.9%	<0.0001
Yield—excl. epilepsy/DEE cohorts	19	25%	18–33%	96.4%	<0.0001
Actionability—excl. epilepsy/DEE cohorts	13	64%	48–78%	96.0%	<0.0001
Actionability—excl. Nakhleh Francis 2023 [56]	15	61%	46–75%	95.4%	<0.0001
**Transformation-robustness analyses—GLMM/logit (** Section 2.5 **)**					
Yield—logit transformation	23	26.84%	20.25–34.65%	96.3%	<0.0001
Yield—GLMM (method.tau = ‘ML’)	23	26.63%	20.15–34.31%	96.3%	<0.0001
Actionability—logit transformation	16	57.66%	43.26–70.86%	90.7%	<0.0001
Actionability—GLMM (method.tau = ‘ML’)	16	61.12%	43.36–76.35%	90.0%	<0.0001

All analyses: random-effects model, Freeman–Tukey double arcsine transformation (except GLMM rows: generalised linear mixed model, method.tau = “ML”), REML estimation of τ^2^. Full annotated version in Supplementary Table S1.

## Data Availability

All data analysed are available in the cited published studies and in the Supplementary Materials. The R code used to perform the meta-analyses and generate the figures is available from the corresponding author upon reasonable request.

## Supplementary Materials

The following supporting information can be downloaded at: https://www.mdpi.com/article/10.3390/ijms27209113/s1.

## Abbreviations

The following abbreviations are used in this manuscript:

ACMG	American College of Medical Genetics and Genomics
aCGH	Array comparative genomic hybridization
ADHD	Attention-deficit/hyperactivity disorder
AI	Artificial intelligence
AMP	Association for Molecular Pathology
ASD	Autism spectrum disorder
CI	Confidence interval
CMA	Chromosomal microarray analysis
CNV	Copy number variant
DD	Developmental delay
DEE	Developmental and epileptic encephalopathy
DOI	Digital object identifier
DSM-5	Diagnostic and Statistical Manual of Mental Disorders, 5th edition
EMR	Electronic medical record
ES	Exome sequencing
FraX	Fragile X syndrome
GDD	Global developmental delay
GLMM	Generalised linear mixed model
GRADE	Grading of Recommendations, Assessment, Development and Evaluations
GS	Genome sequencing
ID	Intellectual disability
JBI	Joanna Briggs Institute
MCA	Multiple congenital anomalies
NDD	Neurodevelopmental disorder
NGS	Next-generation sequencing
PICO	Population, Intervention, Comparison, Outcome
PRISMA	Preferred Reporting Items for Systematic Reviews and Meta-Analyses
PROSPERO	International Prospective Register of Systematic Reviews
REML	Restricted maximum likelihood
VUS	Variant of uncertain significance
WES	Whole-exome sequencing
WGS	Whole-genome sequencing

## References

[1] CDC Developmental Disability Basics. Child Development. https://www.cdc.gov/child-development/about/developmental-disability-basics.html.

[2] Morris-Rosendahl D.J., Crocq M.-A. (2020). Neurodevelopmental disorders-the history and future of a diagnostic concept. Dialogues Clin. Neurosci..

[3] Cainelli E., Bisiacchi P. (2023). Neurodevelopmental disorders: Past, present, and future. Children.

[4] Cumming J., Middleton L., Silwal P., Tenbensel T. (2021). Integrated care in Aotearoa New Zealand 2008–2020. Int. J. Integr. Care.

[5] Tengsujaritkul M., Louthrenoo O., Likhitweerawong N., Boonchooduang N., Srisurapanont M. (2026). Diagnostic and clinical utility of exome sequencing and chromosomal microarray in children with GDD/ID: A meta-analysis. Ann. Med..

[6] Anazi S., Maddirevula S., Faqeih E., Alsedairy H., Alzahrani F., E Shamseldin H., Patel N., Hashem M., Ibrahim N., Abdulwahab F. (2017). Clinical genomics expands the morbid genome of intellectual disability and offers a high diagnostic yield. Mol. Psychiatry.

[7] Zhang J., Xu Y., Liu Y., Yue L., Jin H., Chen Y., Wang D., Wang M., Chen G., Yang L. (2024). Genetic testing for global developmental delay in early childhood. JAMA Netw. Open.

[8] Rodan L.H., Stoler J., Chen E., Geleske T., Council on Genetics (2025). Genetic evaluation of the child with intellectual disability or global developmental delay: Clinical report. Pediatrics.

[9] Shaffer L.G. (2005). American College of Medical Genetics guideline on the cytogenetic evaluation of the individual with developmental delay or mental retardation. Genet. Med..

[10] Lai M.-C., Lombardo M.V., Baron-Cohen S. (2014). Autism. Lancet.

[11] Tick B., Bolton P., Happé F., Rutter M., Rijsdijk F. (2016). Heritability of autism spectrum disorders: A meta-analysis of twin studies. J. Child Psychol. Psychiatry.

[12] He X., Sanders S.J., Liu L., De Rubeis S., Lim E.T., Sutcliffe J.S., Schellenberg G.D., Gibbs R.A., Daly M.J., Buxbaum J.D. (2013). Integrated model of de novo and inherited genetic variants yields greater power to identify risk genes. PLoS Genet..

[13] Hu C., Wang Y., Li C., Mei L., Zhou B., Li D., Li H., Xu Q., Xu X. (2023). Targeted sequencing and clinical strategies in children with autism spectrum disorder: A cohort study. Front. Genet..

[14] Genovese A., Butler M.G. (2020). Clinical assessment, genetics, and treatment approaches in autism spectrum disorder (ASD). Int. J. Mol. Sci..

[15] Sheth F., Shah J., Jain D., Shah S., Patel H., Patel K., Solanki D.I., Iyer A.S., Menghani B., Mhatre P. (2023). Comparative yield of molecular diagnostic algorithms for autism spectrum disorder diagnosis in India: Evidence supporting whole exome sequencing as first tier test. BMC Neurol..

[16] Schaefer G.B., Mendelsohn N.J. (2013). Clinical genetics evaluation in identifying the etiology of autism spectrum disorders: 2013 guideline revisions. Genet. Med..

[17] Selvanayagam T., Hoang N., Sarikaya E., Howe J., Russell C., Iaboni A., Quirbach M., Marshall C.R., Szatmari P., Anagnostou E. (2025). Clinical utility of genome sequencing in autism: Illustrative examples from a genomic research study. J. Med. Genet..

[18] Hoang N., Cytrynbaum C., Scherer S.W. (2018). Communicating complex genomic information: A counselling approach derived from research experience with autism spectrum disorder. Patient Educ. Couns..

[19] Savatt J.M., Myers S.M. (2021). Genetic testing in neurodevelopmental disorders. Front. Pediatr..

[20] Schaefer G.B., Bodensteiner J.B. (1992). Evaluation of the child with idiopathic mental retardation. Pediatr. Clin. N. Am..

[21] Miller D.T., Adam M.P., Aradhya S., Biesecker L.G., Brothman A.R., Carter N.P., Church D.M., Crolla J.A., Eichler E.E., Epstein C.J. (2010). Consensus statement: Chromosomal microarray is a first-tier clinical diagnostic test for individuals with developmental disabilities or congenital anomalies. Am. J. Hum. Genet..

[22] Jang W., Kim Y., Han E., Park J., Chae H., Kwon A., Choi H., Kim J., Son J.-O., Lee S.-J. (2019). Chromosomal microarray analysis as a first-tier clinical diagnostic test in patients with developmental delay/intellectual disability, autism spectrum disorders, and multiple congenital anomalies: A prospective multicenter study in Korea. Ann. Lab. Med..

[23] Srivastava S., Love-Nichols J.A., Dies K.A., Ledbetter D.H., Martin C.L., Chung W.K., Firth H.V., Frazier T., Hansen R.L., Prock L. (2019). Meta-analysis and multidisciplinary consensus statement: Exome sequencing is a first-tier clinical diagnostic test for individuals with neurodevelopmental disorders. Genet. Med..

[24] Fu J.M., Satterstrom F.K., Peng M., Brand H., Collins R.L., Dong S., Wamsley B., Klei L., Wang L., Hao S.P. (2022). Rare coding variation provides insight into the genetic architecture and phenotypic context of autism. Nat. Genet..

[25] Vorstman J.A.S., Parr J.R., Moreno-De-Luca D., Anney R.J.L., Nurnberger J.I., Hallmayer J.F. (2017). Autism genetics: Opportunities and challenges for clinical translation. Nat. Rev. Genet..

[26] Suárez A.S., Menéndez B.M., Escamilla E.E., Sarries F.J.M., Garrido M.I.E., Gil-Fournier B., León S.R., Gribble B.R., Espinosa J.F.Q., Romero A.J.A. (2024). Whole exome sequencing and panel-based analysis in 176 Spanish children with neurodevelopmental disorders: Focus on autism spectrum disorder and/or intellectual disability/global developmental delay. Genes.

[27] Boyarchuk O., Volianska L., Smashna O., Makukh H. (2024). Exome sequencing in 90 children with developmental delay: A single-center experience. Front. Genet..

[28] Arteche-López A., Rodríguez M.J.G., Calvin M.T.S., Quesada-Espinosa J.F., Rosales J.M.L., Milla C.P., Gómez-Manjón I., Mayoral I.H., de la Fuente R.P., de Bustamante A.D. (2021). Towards a change in the diagnostic algorithm of autism spectrum disorders: Evidence supporting whole exome sequencing as a first-tier test. Genes.

[29] Arora K., Balwani M., Wakchaure A., Passi G.R. (2025). Yield of genetic testing in children with autism spectrum disorder-A single-center experience. Ann. Indian Acad. Neurol..

[30] Shchubelka K., Turova L., Wolfsberger W., Kalanquin K., Williston K., Kurutsa O., Makovetska A., Hasynets Y., Mirutenko V., Vakerych M. (2024). Genetic determinants of global developmental delay and intellectual disability in Ukrainian children. J. Neurodev. Disord..

[31] Kyrylova L.H., Miroshnikov O.O., Kharytonov V.I., Dolenko O.O., Badiuk V.M., Dyukareva-Bezdenezhnykh S.V. (2024). Comparative effectiveness of genetic diagnostic methods in children with nervous system disorders in Ukraine. Int. Neurol. J..

[32] Lamilla J., Castro-Cuesta T.A., Rueda-Gaitán P., Pinto L.C.R., Gutiérrez D.A.R., Rubio Y.N.S., Estrada-Serrato C., Londoño O., Rucinski C., Arcos-Burgos M. (2025). A robust and comprehensive study of the molecular and genetic basis of neurodevelopmental delay in a sample of 3244 patients, evaluated by exome analysis in a Latin population. Diagnostics.

[33] Stafford C.F., Sanchez-Lara P.A. (2022). Impact of genetic and genomic testing on the clinical management of patients with autism spectrum disorder. Genes.

[34] Runheim H., Pettersson M., Hammarsjö A., Nordgren A., Henriksson M., Lindstrand A., Levin L.-Å., Johansson Soller M. (2023). The cost-effectiveness of whole genome sequencing in neurodevelopmental disorders. Sci. Rep..

[35] Postma J.K., Harrison M., Kutcher S., Webster R.J., Cloutier M., Bourque D.K., Yu A.C., Carter M.T. (2024). The diagnostic yield of genetic and metabolic investigations in syndromic and nonsyndromic patients with autism spectrum disorder, global developmental delay, or intellectual disability from a dedicated neurodevelopmental disorders genetics clinic. Am. J. Med. Genet. A.

[36] Manickam K., McClain M.R., Demmer L.A., Biswas S., Kearney H.M., Malinowski J., Massingham L.J., Miller D., Yu T.W., Hisama F.M. (2021). Exome and genome sequencing for pediatric patients with congenital anomalies or intellectual disability: An evidence-based clinical guideline of the American College of Medical Genetics and Genomics (ACMG). Genet. Med..

[37] Clark M.M., Stark Z., Farnaes L., Tan T.Y., White S.M., Dimmock D., Kingsmore S.F. (2018). Meta-analysis of the diagnostic and clinical utility of genome and exome sequencing and chromosomal microarray in children with suspected genetic diseases. npj Genom. Med..

[38] El Naofal M., Ramaswamy S., Alsarhan A., Nugud A., Sarfraz F., Janbaz H., Taylor A., Jain R., Halabi N., Yaslam S. (2023). The genomic landscape of rare disorders in the Middle East. Genome Med..

[39] Page M.J., McKenzie J.E., Bossuyt P.M., Boutron I., Hoffmann T.C., Mulrow C.D., Shamseer L., Tetzlaff J.M., Akl E.A., Brennan S.E. (2021). The PRISMA 2020 statement: An updated guideline for reporting systematic reviews. BMJ.

[40] Demos M., Guella I., DeGuzman C., McKenzie M.B., Buerki S.E., Evans D.M., Toyota E.B., Boelman C., Huh L.L., Datta A. (2019). Diagnostic yield and treatment impact of targeted exome sequencing in early-onset epilepsy. Front. Neurol..

[41] Minardi R., Licchetta L., Baroni M.C., Pippucci T., Stipa C., Mostacci B., Severi G., Toni F., Bergonzini L., Carelli V. (2020). Whole-exome sequencing in adult patients with developmental and epileptic encephalopathy: It is never too late. Clin. Genet..

[42] Charouf D., Miller D., Haddad L., White F.A., Boustany R.-M., Obeid M. (2024). High diagnostic yield and clinical utility of next-generation sequencing in children with epilepsy and neurodevelopmental delays: A retrospective study. Int. J. Mol. Sci..

[43] Pagan C., Goubran-Botros H., Delorme R., Benabou M., Lemière N., Murray K., Amsellem F., Callebert J., Chaste P., Jamain S. (2017). Disruption of melatonin synthesis is associated with impaired 14-3-3 and miR-451 levels in patients with autism spectrum disorders. Sci. Rep..

[44] Ji J., Shen L., Bootwalla M., Quindipan C., Tatarinova T., Maglinte D.T., Buckley J., Raca G., Saitta S.C., Biegel J.A. (2019). A semiautomated whole-exome sequencing workflow leads to increased diagnostic yield and identification of novel candidate variants. Cold Spring Harb. Mol. Case Stud..

[45] Wu D., Wu Y., Lan Y., Lan S., Zhong Z., Li D., Zheng Z., Wang H., Ma L. (2023). Chromosomal aberrations in pediatric patients with moderate/severe developmental delay/intellectual disability with abundant phenotypic heterogeneities: A single-center study. Pediatr. Neurol..

[46] Hakizimana O., Hitayezu J., Uyisenga J.P., Dukuze N., Akimana M.V., Mizero L., Bampire C., Mudenge C., Butoto X.K., Helou L. (2026). Clinical utility of trio exome sequencing in Rwandan children with autism spectrum disorder. Mol. Genet. Genom. Med..

[47] Abarca-Barriga H.H., Vásquez Sotomayor F., Punil-Luciano R., Laso-Salazar M.C., Barrón-Pastor H.J. (2025). Identification of intragenic variants in pediatric patients with intellectual disability in Peru. BMC Med. Genom..

[48] Monroe G.R., Frederix G.W., Savelberg S.M.C., de Vries T.I., Duran K.J., van der Smagt J.J., Terhal P.A., van Hasselt P.M., Kroes H.Y., Verhoeven-Duif N.M. (2016). Effectiveness of whole-exome sequencing and costs of the traditional diagnostic trajectory in children with intellectual disability. Genet. Med..

[49] Ilic N., Maric N., Maver A., Armengol L., Kravljanac R., Cirkovic J., Krstic J., Radivojevic D., Cirkovic S., Ostojic S. (2024). Reverse phenotyping after whole-exome sequencing in children with developmental delay/intellectual disability—An exception or a necessity?. Genes.

[50] Medyanik A.D., Anisimova P.E., Kustova A.O., Tarabykin V.S., Kondakova E.V. (2025). Developmental and epileptic encephalopathy: Pathogenesis of intellectual disability beyond channelopathies. Biomolecules.

[51] Raga S., Specchio N., Rheims S., Wilmshurst J.M. (2021). Developmental and epileptic encephalopathies: Recognition and approaches to care. Epileptic Disord..

[52] Shimizu H., Morimoto Y., Yamamoto N., Tayama T., Ozawa H., Imamura A., Czuczwar S.J. (2022). Overlap between epilepsy and neurodevelopmental disorders: Insights from clinical and genetic studies. Epilepsy.

[53] McKnight D., Bristow S.L., Truty R.M., Morales A., Stetler M., Westbrook M.J., Robinson K., Riethmaier D., Borlot F., Kellogg M. (2022). Multigene panel testing in a large cohort of adults with epilepsy. Neurol. Genet..

[54] Koh H.Y., Smith L., Wiltrout K.N., Podury A., Chourasia N., D’Gama A.M., Park M., Knight D., Sexton E.L., Koh J.J. (2023). Utility of exome sequencing for diagnosis in unexplained pediatric-onset epilepsy. JAMA Netw. Open.

[55] Wu F., Ji X.N., Cheng P.D., Zhao S.Z., Feng S., Liu W.T., Chen J.X., Zhang X., Wang H.X., Chen Q. (2026). Diagnostic genetic findings from exome sequencing in a cohort of 1,109 children with epilepsy. Neurol. Genet..

[56] Nakhleh Francis Y., Hershkovitz T., Ekhilevitch N., Habib C., Ravid S., Tal G., Schertz M., Mory A., Zinger A., Baris Feldman H. (2023). Publicly funded exome sequencing for outpatients with neurodevelopmental disorders demonstrates a high rate of unexpected findings impacting medical management. Genet. Med. Open.

[57] Saam J., Gudgeon J., Aston E., Brothman A.R. (2008). How physicians use array comparative genomic hybridization results to guide patient management in children with developmental delay. Genet. Med..

[58] Henderson L.B., Applegate C.D., Wohler E., Sheridan M.B., Hoover-Fong J., Batista D.A.S. (2014). The impact of chromosomal microarray on clinical management: A retrospective analysis. Genet. Med..

[59] Balduzzi S., Rücker G., Schwarzer G. (2019). How to perform a meta-analysis with R: A practical tutorial. Evid. Based Ment. Health.

[60] Freeman M.F., Tukey J.W. (1950). Transformations related to the angular and the square root. Ann. Math. Stat..

[61] Sterne J.A.C., Sutton A.J., Ioannidis J.P.A., Terrin N., Jones D.R., Lau J., Carpenter J., Rücker G., Harbord R.M., Schmid C.H. (2011). Recommendations for examining and interpreting funnel plot asymmetry in meta-analyses of randomised controlled trials. BMJ.

[62] Guyatt G.H., Oxman A.D., Vist G.E., Kunz R., Falck-Ytter Y., Alonso-Coello P., Schünemann H.J. (2008). GRADE: An emerging consensus on rating quality of evidence and strength of recommendations. BMJ.

[63] Xu M., Ji Y., Zhang T., Jiang X., Fan Y., Geng J., Li F. (2018). Clinical application of chromosome microarray analysis in Han Chinese children with neurodevelopmental disorders. Neurosci. Bull..

[64] Coulter M.E., Miller D.T., Harris D.J., Hawley P., Picker J., E Roberts A., Sobeih M.M., Irons M. (2011). Chromosomal microarray testing influences medical management. Genet. Med..

[65] Harris H.K., Sideridis G.D., Barbaresi W.J., Harstad E. (2020). Pathogenic yield of genetic testing in autism spectrum disorder. Pediatrics.

[66] Tao V.Q., Chan K.Y.K., Chu Y.W.Y., Mok G.T.K., Tan T.Y., Yang W., Lee S.L., Tang W.F., Tso W.W.Y., Lau E.T. (2014). The clinical impact of chromosomal microarray on paediatric care in Hong Kong. PLoS ONE.

[67] Stavropoulos D.J., Merico D., Jobling R., Bowdin S., Monfared N., Thiruvahindrapuram B., Nalpathamkalam T., Pellecchia G., Yuen R.K.C., Szego M.J. (2016). Whole-genome sequencing expands diagnostic utility and improves clinical management in paediatric medicine. npj Genom. Med..

[68] Du X., Gao X., Liu X., Shen L., Wang K., Fan Y., Sun Y., Luo X., Liu H., Wang L. (2018). Genetic diagnostic evaluation of trio-based whole exome sequencing among children with diagnosed or suspected autism spectrum disorder. Front. Genet..

[69] Cucinotta F., Lintas C., Tomaiuolo P., Baccarin M., Picinelli C., Castronovo P., Sacco R., Piras I.S., Turriziani L., Ricciardello A. (2023). Diagnostic yield and clinical impact of chromosomal microarray analysis in autism spectrum disorder. Mol. Genet. Genom. Med..

[70] Ma H., Zhu L., Yang X., Ao M., Zhang S., Guo M., Dai X., Ma X., Zhang X. (2024). Genetic and phenotypic analysis of 225 Chinese children with developmental delay and/or intellectual disability using whole-exome sequencing. BMC Genom..

[71] Chang Y.-M., Huang Y.-T., Lai P.-C. (2025). Genetic testing for diagnosing neurodevelopmental disorders and epilepsy: A systematic review and meta-analysis. Syst. Rev..

[72] Bar O., Vahey E., Mintz M., Frye R.E., Boles R.G. (2024). Reanalysis of trio whole-genome sequencing data doubles the yield in autism spectrum disorder: De novo variants present in half. Int. J. Mol. Sci..

[73] Besterman A.D., Adams D.J., Wong N.R., Schneider B.N., Mehta S., DiStefano C., Wilson R.B., Martinez-Agosto J.A., Jeste S.S. (2025). Genomics-informed neuropsychiatric care for neurodevelopmental disorders: Results from a multidisciplinary clinic. Genet. Med..

[74] Children’s Hospital of Philadelphia Dravet Syndrome. https://www.chop.edu/conditions-diseases/dravet-syndrome.

[75] Dilena R., Mauri E., Di Fonzo A., Bana C., Ajmone P.F., Rigamonti C., Catenio T., Gangi S., Striano P., Fumagalli M. (2022). Case report: Effect of targeted therapy with carbamazepine in KCNQ2 neonatal epilepsy. Front. Neurol..

[76] Jefferies N. Diagnostic Odyssey in Rare Disease-Knowledge Hub. GeNotes. https://www.genomicseducation.hee.nhs.uk/genotes/knowledge-hub/the-diagnostic-odyssey-in-rare-disease/.

[77] Sawyer S., Hartley T., Dyment D., Beaulieu C., Schwartzentruber J., Smith A., Bedford H., Bernard G., Bernier F., Brais B. (2016). Utility of whole-exome sequencing for those near the end of the diagnostic odyssey: Time to address gaps in care. Clin. Genet..

[78] Peltekova I., Yusuf A., Frei J., Savion-Lemieux T., Joober R., Howe J., Scherer S.W., Elsabbagh M. (2021). Predictors of empowerment in parents of children with autism and related neurodevelopmental disorders who are undergoing genetic testing. Mol. Genet. Genom. Med..

[79] Blesson A., Cohen J.S. (2020). Genetic counseling in neurodevelopmental disorders. Cold Spring Harb. Perspect. Med..

[80] Hellquist A., Tammimies K. (2022). Access, utilization, and awareness for clinical genetic testing in autism spectrum disorder in Sweden: A survey study. Autism.

[81] Masri A.T., Nasir A., Irshaid F., Alomari F., Irshaid A., Al-Qudah A., Nafi O., Almomani M. (2022). Genetic evaluation of children with autism spectrum disorders in developing and low-resource areas. Autism.

[82] Arcebido K., Tuliao E.V., Ibarra A.M., Russell K., Valdes A., Shinkre S., Gefen S., Evans A., Barella S., Wadei J. (2025). Determining rates of genetic testing in the evaluation of autism spectrum disorder and intellectual and developmental disorder. Autism.

[83] Moreno-De-Luca D., Kavanaugh B.C., Best C.R., Sheinkopf S.J., Phornphutkul C., Morrow E.M. (2020). Clinical genetic testing in autism spectrum disorder in a large community-based population sample. JAMA Psychiatry.

[84] Garrido-Torres N., Rodríguez R.M., Alemany-Navarro M., Sánchez-García J., García-Cerro S., Ayuso M.I., González-Meneses A., Martinez-Mir A., Ruiz-Veguilla M., Crespo-Facorro B. (2024). Exploring genetic testing requests, genetic alterations and clinical associations in a cohort of children with autism spectrum disorder. Eur. Child Adolesc. Psychiatry.

[85] Richards S., Aziz N., Bale S., Bick D., Das S., Gastier-Foster J., Grody W.W., Hegde M., Lyon E., Spector E. (2015). Standards and guidelines for the interpretation of sequence variants: A joint consensus recommendation of the American College of Medical Genetics and Genomics and the Association for Molecular Pathology. Genet. Med..

[86] Carter M.T., Srour M., Au P.-Y.B., Buhas D., Dyack S., Eaton A., Inbar-Feigenberg M., Howley H., Kawamura A., Lewis S.M.E. (2023). Genetic and metabolic investigations for neurodevelopmental disorders: Position statement of the Canadian College of Medical Geneticists (CCMG). J. Med. Genet..

[87] Assadourian A.A., Martinez-Agosto J.A. (2025). Precision diagnostic and therapeutic interventions in rare genetic neurodevelopmental disorders. Pediatr. Res..

[88] Finucane B.M., Ledbetter D.H., Vorstman J.A. (2021). Diagnostic genetic testing for neurodevelopmental psychiatric disorders: Closing the gap between recommendation and clinical implementation. Curr. Opin. Genet. Dev..

[89] Kreiman B.L., Boles R.G. (2020). State of the art of genetic testing for patients with autism: A practical guide for clinicians. Semin. Pediatr. Neurol..

[90] Lee S., Seo G.H., Kim S.Y., Jang S.S., Jang S., Choi S., Chin H., Lee S.J., Oh D.E., Ryu S.W. (2025). Clinical utility of genome sequencing in rare diseases: Lessons from a single-center study of 1,452 Korean families. npj Genom. Med..

[91] Butler M.G., Moreno-De-Luca D., Persico A.M. (2022). Actionable genomics in clinical practice: Paradigmatic case reports of clinical and therapeutic strategies based upon genetic testing. Genes.

[92] Shillington A., Lamy M., Dominick K.C., Sorter M., Erickson C.A., Hopkin R. (2022). A collaborative psychiatric-genetics inpatient care delivery model improves access to clinical genetic evaluation, testing, and diagnosis for patients with neurodevelopmental disorders. Front. Genet..

[93] Wang A., Little I.D., Carter D., Pham S., Piper M., Ramírez-Renta G.M., Telaak S., Gunter C. (2024). Provider-reported experiences, barriers, and perspectives on genetic testing as part of autism diagnosis. PLoS ONE.

[94] Pandey R., Brennan N.F., Trachana K., Katsandres S., Bodamer O., Belmont J., Veenstra D.L., Peng S. (2025). A meta-analysis of diagnostic yield and clinical utility of genome and exome sequencing in pediatric rare and undiagnosed genetic diseases. Genet. Med..

