# Supplementary material for: Diagnostic Yield and Clinical Actionability of Genetic Testing in Neurodevelopmental Disorders: A Systematic Review and Meta-Analysis
Source: Int J Mol Sci. 2026 Oct 14;27(20):9113. doi: 10.3390/ijms27209113 (PMC13705074; doi:10.3390/ijms27209113)
Supplement: Supplementary file 1 [file ijms-27-09113-s001.zip › II PRISMA_2020_Checklist_Dumitra_A.pdf]

PRISMA 2020 Checklist

Diagnostic Yield and Clinical Actionability of Genetic Testing in Neurodevelopmental Disorders: A Systematic Review and Meta-Analysis

Section and Topic	Item #	Checklist item	Location where item is reported
**TITLE**			
**Title**	1	Identify the report as a systematic review.	p. 1 (Title)
**ABSTRACT**			
**Abstract**	2	See the PRISMA 2020 for Abstracts checklist.	p. 1 (structured abstract: Background/Objectives, Methods, Results, Conclusions); not separately checked against the PRISMA 2020 for Abstracts 12-item checklist
**INTRODUCTION**			
**Rationale**	3	Describe the rationale for the review in the context of existing knowledge.	pp. 1–2 (Section 1, Introduction, paragraphs 1–3)
**Objectives**	4	Provide an explicit statement of the objective(s) or question(s) the review addresses.	p. 2 (Section 1, end)
**METHODS**			
**Eligibility criteria**	5	Specify the inclusion and exclusion criteria for the review and how studies were grouped for the syntheses.	pp. 2–3 (Section 2.2); grouping by testing modality for synthesis pre-specified in Section 2.5
**Information sources**	6	Specify all databases, registers, websites, organisations, reference lists and other sources searched or consulted to identify studies. Specify the date when each source was last searched or consulted.	p. 2 (Section 2.1) - PubMed and Web of Science (searched 1 October 2026), an AI-assisted search (Undermind), and reference-list screening of included articles.
**Search strategy**	7	Present the full search strategies for all databases, registers and websites, including any filters and limits used.	p. 2 (Section 2.1); full verbatim strategies in Supplementary Table S5
**Selection process**	8	Specify the methods used to decide whether a study met the inclusion criteria of the review, including how many reviewers screened each record and each report retrieved, whether they worked independently, and if applicable, details of automation tools used in the process.	p. 2–3 (Section 2.2, last sentence) - one investigator performed screening using a standardized form, with a second investigator independently verifying accuracy for all included studies; discrepancies resolved by discussion, with a third investigator consulted where consensus could not be reached
**Data collection process**	9	Specify the methods used to collect data from reports, including how many reviewers collected data from each report, whether they worked independently, any processes for obtaining or confirming data from study investigators, and if applicable, details of automation tools used in the process.	pp. 2–3 (Section 2.2, last sentence)
**Data items**	10a	List and define all outcomes for which data were sought. Specify whether all results that were compatible with each outcome domain in each study were sought (e.g. for all measures, time points, analyses), and if not, the methods used to decide which results to collect.	p. 3 (Section 2.4)
	10b	List and define all other variables for which data were sought (e.g. participant and intervention characteristics, funding sources). Describe any	p. 2 (Section 2.2 - extraction categories); p. 3 (Section 2.5 — no summary statistics missing for primary analyses)

Section and Topic	Item #	Checklist item	Location where item is reported
		assumptions made about any missing or unclear information.	
**Study risk of bias assessment**	11	Specify the methods used to assess risk of bias in the included studies, including details of the tool(s) used, how many reviewers assessed each study and whether they worked independently, and if applicable, details of automation tools used in the process.	p. 3 (Section 2.6)
**Effect measures**	12	Specify for each outcome the effect measure(s) (e.g. risk ratio, mean difference) used in the synthesis or presentation of results.	p. 3 (Section 2.5) - single-group proportions (diagnostic yield, clinical actionability), analysed via metaprop() with Freeman–Tukey double-arcsine transformation
**Synthesis methods**	13a	Describe the processes used to decide which studies were eligible for each synthesis (e.g. tabulating the study intervention characteristics and comparing against the planned groups for each synthesis (item #5)).	p. 3 (Section 2.5 - pre-specified subgroups by modality); Table 2 (Method column)
	13b	Describe any methods required to prepare the data for presentation or synthesis, such as handling of missing summary statistics, or data conversions.	p. 3 (Section 2.5)
	13c	Describe any methods used to tabulate or visually display results of individual studies and syntheses.	Figure 1 p. 4; Table 1 pp. 4–5; Table 2 pp. 5–6; Figures 2–5 pp. 7–9; Table 3 pp. 9–10; Figure 6 p. 10
	13d	Describe any methods used to synthesise results and provide a rationale for the choice(s). If meta-analysis was performed, describe the model(s), method(s) to identify the presence and extent of statistical heterogeneity, and software package(s) used.	p. 3 (Section 2.5) - R v4.5.3, package meta v8.5.0, metaprop(), random-effects model, REML, I^2/Q/τ^2
	13e	Describe any methods used to explore possible causes of heterogeneity among study results (e.g. subgroup analysis, meta-regression).	p. 3 (Section 2.5) - pre-specified subgroup analysis by testing modality, Q-between test
	13f	Describe any sensitivity analyses conducted to assess robustness of the synthesised results.	p. 3 (Section 2.5) - sensitivity analyses restricting to methodologically homogeneous studies (flag=1), excluding Selvanayagam et al. (2025), excluding epilepsy/DEE-associated cohorts, and excluding Nakhleh Francis et al. (2023); robustness additionally assessed via logit-transformed and GLMM estimates and 95% prediction intervals
**Reporting bias assessment**	14	Describe any methods used to assess risk of bias due to missing results in a synthesis (arising from reporting biases).	p. 3 (Section 2.5) - funnel plots and Egger's regression test (threshold $p < 0.1$)
**Certainty assessment**	15	Describe any methods used to assess certainty (or confidence) in the body of evidence for an outcome.	p. 3 (Section 2.5, end) - GRADE approach, adapted for single-group prevalence/proportion meta-analyses
**RESULTS**			
**Study selection**	16a	Describe the results of the search and selection process, from the number of records identified in the search to the number of studies included in the review, ideally using a flow diagram.	p. 4 (Section 3.1, Figure 1)

Section and Topic	Item #	Checklist item	Location where item is reported
	16b	Cite studies that might appear to meet the inclusion criteria, but which were excluded, and explain why they were excluded.	p. 4 (Section 3.1) - 14 studies excluded at full-text; full list with individual reasons in Supplementary Table S6
**Study characteristics**	17	Cite each included study and present its characteristics.	pp. 5–6 (Table 2, condensed); full characteristics in Supplementary Table S2
**Risk of bias in studies**	18	Present assessments of risk of bias for each included study.	pp. 4–5 (Table 1, JBI Critical Appraisal Checklist scores per study)
**Results of individual studies**	19	For all outcomes, present, for each study: (a) summary statistics for each group (where appropriate) and (b) an effect estimate and its precision (e.g. confidence/credible interval), ideally using structured tables or plots.	pp. 5–6 (Table 2, per-study yield/actionability %); pp. 7–8 (Figures 2–3, per-study estimates with 95% CI)
**Results of syntheses**	20a	For each synthesis, briefly summarise the characteristics and risk of bias among contributing studies.	pp. 8–9 (Section 3.4.3)
	20b	Present results of all statistical syntheses conducted. If meta-analysis was done, present for each the summary estimate and its precision (e.g. confidence/credible interval) and measures of statistical heterogeneity. If comparing groups, describe the direction of the effect.	pp. 7–10 (Sections 3.4.1–3.4.3; Table 3)
	20c	Present results of all investigations of possible causes of heterogeneity among study results.	pp. 8–9 (Section 3.4.3 - subgroup-by-modality results, Q-between statistics)
	20d	Present results of all sensitivity analyses conducted to assess the robustness of the synthesised results.	pp. 9–10 (Section 3.4.4; Table 3)
**Reporting biases**	21	Present assessments of risk of bias due to missing results (arising from reporting biases) for each synthesis assessed.	p. 10 (Section 3.4.5, Figure 6 - Egger's test and funnel plots)
**Certainty of evidence**	22	Present assessments of certainty (or confidence) in the body of evidence for each outcome assessed.	p. 10 (Section 3.5); full domain-level ratings in Supplementary Table S4
**DISCUSSION**			
**Discussion**	23a	Provide a general interpretation of the results in the context of other evidence.	pp. 10–12 (Sections 4.1–4.5)
	23b	Discuss any limitations of the evidence included in the review.	pp. 12–13 (Section 4.6)
	23c	Discuss any limitations of the review processes used.	Not reported - Section 4.6 addresses limitations of the included evidence, but does not separately discuss limitations of the review process itself (e.g., restriction to two databases plus manual supplementation, English-language restriction, single-reviewer data-collection form)
	23d	Discuss implications of the results for practice, policy, and future research.	pp. 12–13 (Section 4.6; Section 5, Conclusions - three future-priority points)
**OTHER INFORMATION**			
**Registration and protocol**	24a	Provide registration information for the review, including register name and registration number, or state that the review was not registered.	p. 2 (Section 2.1) - registered with PROSPERO, CRD420261452265

Section and Topic	Item #	Checklist item	Location where item is reported
	24b	Indicate where the review protocol can be accessed, or state that a protocol was not prepared.	p. 2 (Section 2.1) - accessible via the PROSPERO registration record cited above; no separate protocol document referenced
	24c	Describe and explain any amendments to information provided at registration or in the protocol.	An amendment was made to the PROSPERO record (Version 2.0); see PROSPERO record for details
**Support**	25	Describe sources of financial or non-financial support for the review, and the role of the funders or sponsors in the review.	p. 13 (Funding statement) - funding source and project/grant identifiers reported; the specific role of the funder in the design, conduct, analysis, or reporting of the review is not stated
**Competing interests**	26	Declare any competing interests of review authors.	p. 13 (Conflicts of Interest statement)
**Availability of data, code and other materials**	27	Report which of the following are publicly available and where they can be found: template data collection forms; data extracted from included studies; data used for all analyses; analytic code; any other materials used in the review.	p. 13 (Data Availability Statement) - data and R analytic code addressed; availability of the standardized data-extraction form is not explicitly stated

From: Page MJ, McKenzie JE, Bossuyt PM, Boutron I, Hoffmann TC, Mulrow CD, et al. The PRISMA 2020 statement: an updated guideline for reporting systematic reviews. BMJ 2021;372:n71. doi: 10.1136/bmj.n71. This work is licensed under CC BY 4.0. To view a copy of this license, visit https://creativecommons.org/licenses/by/4.0/
