# Supplementary material for: Diagnostic Yield and Clinical Actionability of Genetic Testing in Neurodevelopmental Disorders: A Systematic Review and Meta-Analysis
Source: Int J Mol Sci. 2026 Oct 14;27(20):9113. doi: 10.3390/ijms27209113 (PMC13705074; doi:10.3390/ijms27209113)
Supplement: Supplementary file 1 [file ijms-27-09113-s001.zip › PROSPERO.NDD.pdf]

NIHR | National Institute for Health and Care Research

PROSPERO
International prospective register of systematic reviews

Diagnostic Yield and Clinical Actionability of Genetic Testing in Neurodevelopmental Disorders: A Systematic Review and Meta-Analysis

Alexandra Aurora Dumitra, Ioana Streață, Adina Turcu-Știolică

REVIEW TITLE AND BASIC DETAILS

Review title

Diagnostic Yield and Clinical Actionability of Genetic Testing in Neurodevelopmental Disorders: A Systematic Review and Meta-Analysis

Condition or domain being studied

Neurodevelopmental Disorder; Autism Spectrum Disorder; Developmental Delay; Intellectual Disability

This review focuses on patients with neurodevelopmental disorders (NDDs), including autism spectrum disorder (ASD), intellectual disability (ID), and global developmental delay (GDD) as primary populations. Two studies investigating developmental and epileptic encephalopathy (DEE) were also included on the basis of substantial genetic and phenotypic overlap with ASD and ID, and current ACMG guidance recommending exome/genome sequencing for ID/DD irrespective of seizure comorbidity. Despite inclusion of ADHD and developmental language disorder as search terms, no eligible studies with these as primary cohort diagnoses were identified.

Rationale for the review

Genetic testing is increasingly central to the evaluation of neurodevelopmental disorders (NDDs), including autism spectrum disorder, intellectual disability, and global developmental delay. However, most existing literature emphasises diagnostic yield alone, with limited quantitative evidence on whether genetic findings actually translate into changes in clinical management (clinical actionability). Prior reviews have noted that few studies report concrete management change, long-term outcomes, or cost-efficiency following genetic diagnosis. This systematic review and meta-analysis addresses this gap by jointly quantifying pooled diagnostic yield and clinical actionability across published NDD cohorts, with pre-specified subgroup analyses by testing modality (chromosomal microarray, whole-exome sequencing, whole-genome sequencing) and sensitivity analyses accounting for methodological heterogeneity. By moving beyond yield alone to assess whether genetic diagnoses prompt specific, attributable

clinical actions, this review aims to clarify the practical clinical utility of genetic testing in NDDs and inform evidence-based testing strategies.

Review objectives

This systematic review and meta-analysis aims to quantify the pooled diagnostic yield and clinical actionability of genetic testing in patients with neurodevelopmental disorders (NDDs), with a primary focus on autism spectrum disorder, intellectual disability, and global developmental delay. Specifically, it examines the extent to which frontline genetic testing — chromosomal microarray, whole-exome sequencing, and whole-genome sequencing — yields clinically actionable outcomes, as measured by diagnostic yield, pharmacological modifications, specialist referrals, and other management changes attributable to a confirmed genetic diagnosis. As no included study contained an untreated control group, the comparative component of the review is addressed indirectly through the reported rate of clinical management change following genetic diagnosis, rather than through direct comparison with untested patients.

Keywords

Neurodevelopmental disorders; Autism spectrum disorder; Intellectual disability; Genetic testing; Diagnostic yield; Whole-exome sequencing; Chromosomal Microarray; Actionability

Country

Romania

ELIGIBILITY CRITERIA

Population 1 change

Included

The inclusion criteria are studies with patients diagnosed with a neurodevelopmental disorder (NDD), with a primary focus on autism spectrum disorder (ASD), intellectual disability (ID), developmental delay (DD), and global developmental delay (GDD). Patients with developmental and epileptic encephalopathy (DEE) co-occurring with ASD, ID, DD, or GDD are eligible, given the substantial genetic and phenotypic overlap between DEE and ASD/ID and current ACMG guidance recommending exome/genome sequencing for ID/DD irrespective of seizure comorbidity.

Excluded

The exclusion criteria are studies enrolling epilepsy or developmental and epileptic encephalopathy (DEE) cohorts without co-occurring ASD, ID, DD, or GDD; case reports with fewer than 5 patients.

Intervention(s) or exposure(s) 1 change

Included

Genetic testing

Genetic/genomic testing performed as part of the diagnostic work-up for a neurodevelopmental disorder, including chromosomal microarray (CMA), whole-exome sequencing (WES, including trio-based WES), and whole-genome sequencing (WGS). Eligible studies must report diagnostic yield and/or clinical actionability — specialist referral, targeted investigation,

medication initiation or contraindication, surgical/procedural intervention, or structured surveillance — directly attributable to the genetic finding.

Excluded

The intervention(s) or exposure(s) exclusion criteria are: studies reporting only technical variant data (e.g., variant frequency or classification) without any post-diagnostic clinical management outcome.

Comparator(s) or control(s)

This review does not have any comparators

Study design

Only nonrandomized study types will be included.

Included

Retrospective and prospective cohort studies, chart reviews, and cross-sectional or pilot studies reporting diagnostic yield and/or clinical actionability of genetic testing in patients with neurodevelopmental disorders.

Excluded

Reviews, guidelines, or narrative summaries without primary data; studies reporting only technical variant data without post-diagnostic clinical outcomes; non-English-language publications; and case reports with fewer than 5 patients.

Context 1 change

Studies conducted in clinical genetics, developmental paediatrics, or diagnostic laboratory settings in which genetic testing was performed as part of the evaluation of neurodevelopmental disorders. No restriction was applied by country or healthcare system. Eligible studies were published in English, identified through PubMed, Web of Science, and an AI-assisted literature search tool (Undermind). A publication-date filter (20 November 2015 onward) was applied to the PubMed and Web of Science database searches.

TIMELINE OF THE REVIEW

Date of first submission to PROSPERO

17 July 2026

Review timeline

Review start date: 1 May 2026. Review end date: 31 October 2026.

Date of registration in PROSPERO

17 July 2026

AVAILABILITY OF FULL PROTOCOL

Availability of full protocol

A full protocol has not been written.

SEARCHING AND SCREENING

Search for unpublished studies
Only published studies will be sought.

Main sources that will be searched 1 change
The main sources to be searched are *PubMed*.

Other sources that will be searched
Web of Science

Search language restrictions 1 change
The review will only include studies published in English.

Search date restrictions
There are no search date restrictions.

Other methods of identifying studies
Other studies will be identified by: *looking through all the articles that cite the papers included in the review ("snowballing" or forward citation searching)* and *searching conference proceedings*.

Link to search strategy
A full search strategy is not available.

Selection process
Studies will be screened independently by at least two people (or person/machine combination) with a process to resolve differences.

Other relevant information about searching and screening
None

DATA COLLECTION PROCESS

Data extraction from published articles and reports
Data will be extracted by one person (or a machine) and checked by at least one other person.

Authors will be asked to provide any required data not available in published reports.

Study risk of bias or quality assessment
Risk of bias will be assessed using: *JBI tools*

Data will be assessed by one person (or a machine) and checked by at least one other person.

Additional information will **not** be sought from study investigators if required information is unclear or unavailable in the study publications/reports.

Reporting bias assessment 1 change
Risk of bias due to missing results will be assessed visually using funnel plots and formally using Egger's regression test (threshold $p < 0.1$), performed separately for diagnostic yield and clinical actionability.

Certainty assessment 1 change
Certainty of findings will be assessed using the GRADE (Grading of Recommendations Assessment, Development and Evaluation) approach, adapted for single-group prevalence meta-analysis, across the domains of risk of bias (informed by JBI Critical Appraisal Checklist

scores), inconsistency (I^2 statistic), indirectness (non-standard outcome definitions across a subset of studies), imprecision (confidence interval width), and publication bias (Egger's regression test), performed separately for diagnostic yield and clinical actionability.

OUTCOMES TO BE ANALYSED

Main outcomes

The two primary outcomes are diagnostic yield and clinical actionability. Diagnostic yield is defined as the proportion of tested patients receiving a confirmed pathogenic or likely pathogenic genetic diagnosis, assessed at the time of test reporting. Clinical actionability is defined as the proportion of patients with a confirmed genetic diagnosis who received at least one specific management recommendation directly attributable to that finding — specialist referral, targeted investigation, medication initiation or contraindication, surgical or procedural intervention, or structured surveillance. Both outcomes will be synthesised as pooled single-group proportions with 95% confidence intervals, using random-effects meta-analysis with the Freeman–Tukey double arcsine transformation.

Additional outcomes

There are no additional outcomes.

PLANNED DATA SYNTHESIS

Strategy for data synthesis 1 change

Diagnostic yield and clinical actionability will each be synthesised separately using meta-analysis of single proportions in R (v4.5.3) with the meta package (v8.5.0). The metaprop() function will be used with the Freeman–Tukey double arcsine transformation (sm = 'PFT') to stabilise variance for proportions approaching 0% or 100%. A random-effects model will be specified a priori given expected differences in population, testing modality, and diagnostic era, with between-study variance (τ^2) estimated by restricted maximum likelihood (REML). Heterogeneity will be quantified using I^2, the Cochran Q statistic, and τ^2. Pre-specified subgroup analyses will compare pooled estimates by testing modality (chromosomal microarray, whole-exome sequencing, whole-genome sequencing), with between-subgroup differences evaluated using the Q-between test. Sensitivity analyses will include: restriction to methodologically homogeneous studies; exclusion of a study using a non-standard actionability framework; exclusion of epilepsy/DEE-associated cohorts; and exclusion of a study with a non-aggregable actionability definition. Analyses using logit-transformed proportions and a generalised linear mixed model (GLMM; method.tau = 'ML') will additionally be performed to assess robustness to the choice of variance-stabilising transformation, alongside 95% prediction intervals to characterise the range of effects expected in a new clinical setting given the anticipated heterogeneity. Comparative effect-size estimation will not be performed, as no included study contains an untreated control group; instead, the comparative component will be addressed through the reported rate of clinical management change following genetic diagnosis.

CURRENT REVIEW STAGE

Stage of the review at this submission

Review stage	Started	Completed
Pilot work	✓	
Formal searching/study identification	✓	
Screening search results against inclusion criteria		
Data extraction or receipt of IPD		
Risk of bias/quality assessment		
Data synthesis		

Review status

The review is currently planned or ongoing.

Publication of review results

Results of the review will be published in English.

REVIEW AFFILIATION, FUNDING AND PEER REVIEW

Review team members

Dr Alexandra Aurora Dumitra (review guarantor and contact) ORCID: 0009-0009-2363-7315. University of Medicine and Pharmacy of Craiova, Romania. Romania.

No conflict of interest declared.

Associate Professor Ioana Streață. University of Medicine and Pharmacy of Craiova, Romania. Romania.

No conflict of interest declared.

Professor Adina Turcu-Știolică. University of Medicine and Pharmacy of Craiova, Romania. Romania.

No conflict of interest declared.

Named contact

Dr Alexandra Aurora Dumitra (alexandra.dumitra@umfcv.ro). ORCID: 0009-0009-2363-7315. University of Medicine and Pharmacy of Craiova, Romania. Romania.

Review affiliation

University of Medicine and Pharmacy of Craiova, Romania

Funding source

Review has no specific/external funding but is supported by guarantor/review team (non-commercial) institutions.

Peer review

There has been no peer review of this planned review.

ADDITIONAL INFORMATION

Review conflict of interest

Declared individual interests are recorded under team member details.. No additional interests are recorded for this review.

Medical Subject Headings

Autism Spectrum Disorder; Exome; Exome Sequencing; Genetic Testing; Intellectual Disability; Neurodevelopmental Disorders; Developmental Disabilities; Whole Genome Sequencing; Microarray Analysis

Revision note 1 change

This revision corrects several fields to accurately reflect the review's eligibility criteria, search methods, and data-synthesis plan. Search sources and date restrictions have been corrected, population criteria have been clarified and expanded, the comparator and language-restriction fields have been aligned with the review's eligibility framework, and the data-synthesis plan has been updated with the correct software version and a fuller list of sensitivity analyses.

SIMILAR REVIEWS

Check for similar records already in PROSPERO

PROSPERO identified a number of existing PROSPERO records that were similar to this one (last check made on 15 July 2026). These are shown below along with the reasons given by that the review team for the reviews being different and/or proceeding.

- Genetic Disorders Associated with Neurodevelopmental Disorders in Children in Saudi Arabia: A Systematic Review and Meta-Analysis [published 11 December 2025] [CRD420251250437]. The review was judged **not to be similar**
- How Often Does Sequencing Give Answers? A Systematic Review and Meta-Analysis of Exome and Genome Sequencing Yield in Children with Congenital Heart Disease [published 17 March 2026] [CRD420261342389]. The review was judged **not to be similar**
- The diagnostic yield of genetic testing for schizophrenia spectrum disorders: a systematic review and meta-analysis [published 5 April 2023] [CRD42023409096]. The review was judged **not to be similar**
- Diagnostic yield and genetic spectrum of monogenic disorders in fetuses with increased nuchal translucency: a systematic review and meta-analysis [published 6 June 2026] [CRD420261414779]. The review was judged **not to be similar**

PROSPERO version history 1 change

- Version 2.0, published 01 Oct 2026
- Version 1.0, published 17 Jul 2026

Disclaimer

The content of this record displays the information provided by the review team. PROSPERO does not peer review registration records or endorse their content.

PROSPERO accepts and posts the information provided in good faith; responsibility for record content rests with the review team. The guarantor for this record has affirmed that the information provided is truthful and that they understand that deliberate provision of inaccurate information may be construed as scientific misconduct.

PROSPERO does not accept any liability for the content provided in this record or for its use. Readers use the information provided in this record at their own risk.

Any enquiries about the record should be referred to the named review contact
