# Supplementary material for: Diagnostic Yield and Clinical Actionability of Genetic Testing in Neurodevelopmental Disorders: A Systematic Review and Meta-Analysis
Source: Int J Mol Sci. 2026 Oct 14;27(20):9113. doi: 10.3390/ijms27209113 (PMC13705074; doi:10.3390/ijms27209113)
Supplement: Supplementary file 1 [file ijms-27-09113-s001.zip › Supplementary_Materials_Dumitra_track.pdf]

Supplementary Materials

Diagnostic Yield and Clinical Actionability of Genetic Testing in Neurodevelopmental Disorders: A Systematic Review and Meta-Analysis

Table S1. Full Sensitivity-Analysis Annotations

Analysis	n	N	Estimate	95% CI	I²	p	Notes
DIAGNOSTIC YIELD							
Primary analysis	23	7,641	28%	21–35%	96.1%	<0.0001	All 23 data points across 22 studies (Charouf et al., 2024 contributes two data points, reported separately for WES and WGS)
Sensitivity - flag=1 (methodologically homogeneous)	15	-	24%	16–33%	96.2%	<0.0001	Excl. Jang 2019, Saam 2008, Demos 2019, Selvanayagam 2025, Minardi 2020, Ji 2019, Charouf 2024 (WES+WGS)
Sensitivity - epilepsy/DEE cohorts excluded	19	-	25%	18–33%	96.4%	<0.0001	Excl. Demos 2019, Minardi 2020, Charouf 2024 (WES+WGS)
Sensitivity - logit transformation	23	-	26.84%	20.25–34.65%	96.3%	<0.0001	Sensitivity analysis using logit-transformed proportions; method='Inverse', sm='PLOGIT'
Sensitivity - GLMM (method.tau='ML')	23	-	26.63%	20.15–34.31%	96.3%	<0.0001	Sensitivity analysis using a generalised linear mixed model; confirms robustness to transformation choice
Subgroup: CMA studies	9	6,190	16%	12–21%	87.6%	<0.0001	Xu 2018, Jang 2019, Henderson 2014, Coulter 2011, Saam 2008, Harris 2020, Tao 2014, Wu 2023, Cucinotta 2023
Subgroup: WES studies	11	1,142	36%	24–48%	91.2%	<0.0001	Demos 2019, Ji 2019, Du 2018, Minardi 2020, Nakhleh Francis 2023, Hakizimana 2026, Charouf 2024 (WES), Abarca-Barriga 2025, Ma 2024, Monroe 2016, Ilic 2024
Subgroup: WGS studies	3	309	43%	30–57%	71.4%	0.0305	Selvanayagam 2025, Stavropoulos 2016, Charouf 2024 (WGS)
Q-between subgroups (yield)	-	-	-	-	-	<0.0001	χ^2=24.73, df=2, p<0.0001 - statistically significant gradient CMA<WES<WGS
Prediction interval (95%)	23	-	-	PI: 2–67%	96.1%	-	Substantially wider than 95% CI (21-35%), reflecting the high observed heterogeneity
CLINICAL ACTIONABILITY							
Primary analysis	16	1,538	58%	44–72%	95.7%	<0.0001	Ji 2019, Wu 2023, Hakizimana 2026, Abarca-Barriga 2025, Monroe 2016, Ilic 2024 excluded (no formal actionability fraction)
Sensitivity - flag=1 (methodologically homogeneous)	9	-	59%	43–74%	91.3%	<0.0001	Excl. Jang 2019, Saam 2008, Henderson 2014, Selvanayagam 2025, Demos 2019, Minardi 2020, Nakhleh Francis 2023, Charouf 2024
Sensitivity - Selvanayagam 2025 excluded	15	-	54%	42–66%	92.9%	<0.0001	Selvanayagam 2025 used broad 3-category framework; contributes ~4pp to primary estimate
Sensitivity - epilepsy/DEE cohorts excluded	13	-	64%	48–78%	96.0%	<0.0001	Excl. Demos 2019, Minardi 2020, Charouf 2024 (combined)
Sensitivity - Nakhleh Francis 2023 excluded	15	-	61%	46–75%	95.4%	<0.0001	Nakhleh Francis actionability = referral-only component of a non-aggregable multi-category framework (see Table S2 notes); minimal impact confirms robustness of retention decision
Sensitivity - logit transformation	16	-	57.66%	43.26–70.86%	90.7%	<0.0001	Sensitivity analysis using logit-transformed proportions; method='Inverse', sm='PLOGIT'

Analysis	n	N	Estimate	95% CI	I²	p	Notes
Sensitivity - GLMM (method.tau='ML')	16	-	61.12%	43.36–76.35%	90.0%	<0.0001	Sensitivity analysis using a generalised linear mixed model
Subgroup: CMA studies	8	1,119	66%	51–80%	92.7%	<0.0001	Xu 2018, Jang 2019, Henderson 2014, Coulter 2011, Saam 2008, Harris 2020, Tao 2014, Cucinotta 2023
Subgroup: WES studies	5	254	40%	30–51%	54.8%	0.0652	Demos 2019, Du 2018, Minardi 2020, Nakhleh Francis 2023, Ma 2024. I²=54.8% - lower heterogeneity within WES subgroup; lower actionability reflects epilepsy/second-tier cohort composition
Subgroup: WGS studies	2	134	80%	6–100%	98.7%	<0.0001	Selvanayagam 2025, Stavropoulos 2016. Charouf 2024 WGS excluded (combined WES+WGS actionability, not separable - Section 2.3). CI uninterpretable
Q-between subgroups (actionability)	-	-	-	-	-	0.0194	χ²=7.88, df=2, p=0.0194 - significant, yield/actionability dissociation: CMA actionability > WES despite lower yield
Prediction interval (95%)	16	-	-	PI: 5–100%	95.7%	-	Substantially wider than 95% CI (44-72%), reflecting the high observed heterogeneity
PUBLICATION BIAS							
Egger's test - diagnostic yield	23	-	-	-	-	0.0008	t=3.94, df=21. Asymmetry significant; likely attributable to structural heterogeneity (WGS/WES studies: higher yield + smaller N), not publication bias per se
Egger's test - clinical actionability	16	-	-	-	-	0.3307	t=-1.01, df=14. No evidence of funnel plot asymmetry

*Referenced in the main text at Section 3.4.4. Full annotated expansion of Table 3 (main text), including per-analysis notes.*Notes: flag=1 = methodologically homogeneous studies. Epilepsy/DEE cohorts = Demos et al. (2019), Minardi et al. (2020), Charouf et al. (2024, WES+WGS arms). Selvanayagam et al. (2025): actionability=100% reflects broad 3-category framework (medical+reproductive+psychosocial) - not directly comparable. Nakhleh Francis et al. (2023): actionability reflects referral-only component of a non-aggregable 4-category framework. Charouf et al. (2024): actionability reported only combined across WES+WGS, excluded from actionability subgroup analysis. All analyses: random-effects model, Freeman–Tukey double arcsine transformation (sm='PFT') unless otherwise noted; GLMM rows use method='GLMM', method.tau='ML'. REML estimation. R v4.5.3, meta package v8.5.0.

Table S2. Full Study Characteristics

Referenced in the main text at Section 3.3, as the full expansion of Table 2.

Full expansion of Table 2 (main text), Section 3.3.

Study	Design	Population (N, Age, Diagnosis)	Method	Dominant Variant Type	Yield %	Action. %	Key Outcome	Sens. Flag	NDD Category	Flag Notes / Denominator Decisions
Xu 2018	Retrospective analysis	434 Han Chinese; 4mo–17yrs; DD/ID & ASD	CMA (CytoScan HD)	Recurrent CNVs (e.g., 7q11.23, 15q11–q13)	13.6%	94.9%	Earlier diagnosis, optimized management, medication optimization, prenatal counseling	Flag 1 - main analysis	Mixed NDD	-
Jang 2019	Prospective multicenter	617 pts (77% 0–5yrs); DD/ID, ASD, MCA	CMA (w/ G-banding)	Abnormal CNVs (65 pathogenic, 57 VPS)	19.8%	71.4% (overall)	Yield > karyotype (19.8% vs 6.2%); 1,663 clinical strategies prompted	Flag 2 - sensitivity only	Mixed NDD	denominator = 581 (all patients with documented follow-up, all variant classes integrated; 36/617 without follow-up excluded). VPS actionability (86%) paradoxically > pathogenic actionability (83.3%); overall

Study	Design	Population (N, Age, Diagnosis)	Method	Dominant Variant Type	Yield %	Action. %	Key Outcome	Sens. Flag	NDD Category	Flag Notes / Denominator Decisions
										415/581 = 71.4% used as primary figure.
Henderson 2014	Retrospective analysis (EMR review)	1,780 patients (median 4.7yrs); MCAs, DD/ID, autism	SNP-based CMA	Pathogenic copy-number changes (CNCs)	12.7%	54.5% (abnormal cohort)	Abnormal CMA results directly prompted management changes in majority of cases	Flag 1 - main analysis	Mixed NDD	denominator = 187 (EMR follow-up documented only; 40/227 patients without follow-up notes excluded - consistent with paper's own primary analysis of 102/187 = 54.5%).
Coulter 2011	Retrospective chart review	N=1,792 children (DD/ID, ASD, congenital anomalies)	Chromosomal microarray (CMA)	Abnormal CNVs (7.3%) & VPS (5.8%)	13.1%	54% (abnormal); 34% (VPS)	CMA as first-tier test materially alters clinical management and reduces redundant work-ups	Flag 1 - main analysis	Mixed NDD	-
Saam 2008	Pilot study / Physician survey	48 children (normal karyotype) w/ DD/MR	aCGH (BAC array)	Copy number alterations (deletions/ duplications)	17.6%	70.8%	Improved management via recurrence counseling, reduced testing, service access	Flag 2 - sensitivity only	DD-ID	actionability ascertained via physician telephone interviews (48/87 positive patients, 55% response rate).
Demos 2019	Cohort, classified by epilepsy duration at WES	180 children (≤5yrs) with unexplained early-onset epilepsy	Targeted WES (620 genes)	Autosomal-dominant	33% (44% for EE)	39% of diagnosed (13% of total)	Reduced time to diagnosis (145 vs 2,882 days); cost savings ($1,290–$5,110 per patient)	Flag 2 - sensitivity only	Epileptic Encephalopathy	epilepsy cohort - included with pre-specified sensitivity analysis excluding this study.
Selvanayagam 2025	Research cohort; retrospective review of returned results	N=202; mean age 10yrs 2mo; ASD	Whole-genome sequencing (WGS)	De novo (LoF, Haploinsufficiency)	49% (100/202)*	100%†	Informed clinical management, ended diagnostic odysseys, facilitated precision-medicine research	Flag 2 - sensitivity only	ASD	research return-of-results protocol (*yield) and broad 3-category actionability framework (†actionability: medical + reproductive + psychosocial).
Harris 2020	Retrospective chart review	500 toddlers (18–36mo) with DSM-5 ASD	CMA and Fragile X	15q deletions/duplications	12.0%	72.2%	Routine genetic testing recommended for all toddlers with ASD to inform clinical management	Flag 1 - main analysis	ASD	-
Tao 2014	Retrospective medical-record review	327 pediatric; ID/DD, ASD, MCAs	NimbleGen 135k oligonucleotide array	Deletions (64.4% of significant)	11.3%	75.7% (significant); 8.6% (total)	28/37 (75.7%) significant findings prompted management changes; supported by Level 1–2 evidence	Flag 1 - main analysis	Mixed NDD	-
Ji 2019	Semiautomated phenotype-driven WES	106 pediatric patients, median 3yrs, suspected	WES (DRAGEN + Exomiser)	Autosomal dominant (52.5%)	36% overall	Yield only‡	Rapid diagnosis; novel candidate genes	Yield only (no actionability	Mixed NDD	included in yield only - no formal actionability fraction reported; ‡ qualitative case descriptions only.

Study	Design	Population (N, Age, Diagnosis)	Method	Dominant Variant Type	Yield %	Action. %	Key Outcome	Sens. Flag	NDD Category	Flag Notes / Denominator Decisions
		genetic disorders					identified; improved critical care management	reported)		
Stavropoulos 2016	Prospective cohort	100 paediatric patients (<18yrs, mean 5.5) referred for genetics	WGS (vs. standard CMA/targeted testing)	Autosomal dominant (63% of sequence-level variants)	34%	38%	Four-fold yield increase vs. CMA (8%); enables faster, actionable diagnosis	Flag 1 - main analysis	Mixed NDD	-
Du 2018	Trio-based WES after negative CMA	80 children (4mo–13yrs); 65 diagnosed ASD, 15 suspected ASD	Trio-based WES	De novo loss-of-function (LOF) (85.7%)	8.8% overall	57% (4/7 diagnosed)	Precision care, systemic screening, reproductive counseling for CMA-negative ASD patients	Flag 1 - main analysis	ASD	-
Minardi 2020	Cohort (adults with DEE)	71 adults (mean 41yrs) with unknown etiology DEE	Whole-exome sequencing (WES)	Autosomal dominant, recessive, X-linked	25.3%	50%	Resolved diagnostic odyssey (median delay 38.7yrs); impacted management in 50% of diagnosed	Flag 2 - sensitivity only	Epileptic Encephalopathy	epilepsy cohort (adult DEE) - included with pre-specified sensitivity analysis excluding this study.
Pagan 2017	Cross-sectional observational	239 ASD, 303 parents, 78 sibs, 278 controls	Targeted gene sequencing; microduplication genotyping	Missense, nonsense, splice-site; ASMT duplication	4.7% (AANAT/ASMT); p=0.56 vs controls	- (not applicable)	No genotype-directed management changes; melatonin as biochemical target regardless of variant status	EXCLUDED (qualitative synthesis only)	ASD	EXCLUDED from quantitative synthesis - qualitative narrative review only. No significant AANAT/ASMT enrichment in ASD vs controls (p=0.56); no actionability documented.
Wu et al., 2023	Retrospective cohort	122 children; DD/ID moderate–severe, abundant phenotypic heterogeneity	CMA	Diverse pathogenic CNVs	37.7% (46/122)	— (yield only)	CMA identified clinically significant CNVs across heterogeneous DD/ID phenotypes	Flag 1 - main analysis	Mixed NDD	No formal actionability fraction reported. 46/122=37.7% confirmed direct from Results section (full-text verified).
Cucinotta et al., 2023	Cohort (recruited at a tertiary service)	329 children; ASD	CMA (aCGH)	Pathogenic /likely pathogenic CNVs	15.2% (50/329) (+27.1% VUS)	26.0% (13/50)	aCGH led to additional medical investigations in 13/50 P/LP cases (25 prescriptions); 48.0% positive outcome	Flag 1 - main analysis	ASD	Full-text verified (Section 3.3 'Array-CGH and clinical management'): "in 13/50 patients (26.0%), the outcome of the array-CGH led to prescribe additional medical exams… Positive outcomes were obtained in 12/25 (48.0%)".
Nakhleh Francis et al., 2023	Retrospective analysis of a national pilot-program cohort	170 children; GDD, publicly funded program	Trio-ES	Diverse diagnostic variants	43.5% (74/170)	27.0% (20/74, referral component)§	4 non-aggregable actionability categories: 9% active management, 19% surveillance, 23% family-focused, 27% referral	Flag 1 - main analysis (yield); Flag 2 - actionability (non-aggregable	GDD	All 4 categories share denominator=74 (diagnosed), confirmed full-text. Categories NOT mutually exclusive; no combined 'any action' figure reported by authors. Referral (largest, best-defined category, 20/74) used as primary actionability estimate; sensitivity analysis

Study	Design	Population (N, Age, Diagnosis)	Method	Dominant Variant Type	Yield %	Action. %	Key Outcome	Sens. Flag	NDD Category	Flag Notes / Denominator Decisions
								frame work)		excluding this study performed (Table S1).
Hakizimana et al., 2026	Retrospective, hospital-based	31 children; ASD (+ID comorbid 96.8%), Rwanda	Trio-WES	Likely pathogenic variants + phenotype-concordant VUS	12.9% (4/31) (29.0% incl. VUS)	— (yield only)	Demonstrated feasibility of trio-WES in a resource-limited African clinical genetics setting	Flag 1 - main analysis	ASD	No formal actionability fraction reported. Full-text verified (PMC13576637, Results): 4/31=12.9% strict LP; 9/31=29.0% incl. VUS concordant with phenotype.
Charouf et al., 2024 (WES)	Retrospective cohort	38 children (WES arm; 49 children underwent NGS, yield reported for 45); epilepsy + NDD, Lebanon	WES	Diverse (incl. FOLR1, QDPR, GRIN2B, SCN1A)	71.1% (27/38)	19.4% (6/31, combined WES+WGS)¶	Major, potentially life-saving medical management changes (medication/dietary) in 6/31 diagnosed children (combined across both arms)	Flag 2 - sensitivity only (epilepsy)	Epileptic Encephalopathy	Source reports that 49 children underwent NGS, with yield calculated for the 45 who underwent WES (n=38) or WGS (n=7); first-line testing (children with prior genetic testing were excluded). Actionability reported ONLY combined across both modalities in source article (6/31=19.4%); cannot be split by modality. The fraction counts etiology-specific treatment changes only (anti-seizure medication or dietary therapy); the 15 patients who required specialist referral are not included.
Charouf et al., 2024 (WGS)	Retrospective cohort	7 children (WGS arm; 49 children underwent NGS, yield reported for 45); epilepsy + NDD, Lebanon	WGS	Diverse (incl. FOLR1, QDPR, GRIN2B, SCN1A)	57.1% (4/7)	19.4% (6/31, combined WES+WGS)¶	Major, potentially life-saving medical management changes (medication/dietary) in 6/31 diagnosed children (combined across both arms)	Flag 2 - sensitivity only (epilepsy)	Epileptic Encephalopathy	Source reports that 49 children underwent NGS, with yield calculated for the 45 who underwent WES (n=38) or WGS (n=7); first-line testing (children with prior genetic testing were excluded). Actionability reported ONLY combined across both modalities in source article (6/31=19.4%); cannot be split by modality. The fraction counts etiology-specific treatment changes only (anti-seizure medication or dietary therapy); the 15 patients who required specialist referral are not included.
Abarca-Barriga et al., 2025	Descriptive cross-sectional (convenience sampling)	124 children; ID, Peru	WES	Pathogenic /likely pathogenic variants	30.6% (38/124)	— (yield only)	Demonstrated diagnostic value of WES in a Peruvian pediatric ID population; 2/10 WES-negative patients positive on subsequent CMA	Flag 1 - main analysis	ID	No formal actionability fraction reported. Full-text verified (Results, 'Variant distribution and classification'): 38/124=30.6% P/LP. Authors explicitly state non-probabilistic convenience sampling and no formal power analysis performed.
Ma et al., 2024	Retrospective cohort	225 children (208/225 trio); DD/ID, China	WES	Diverse pathogenic variants	42.67% (96/225)	46.9% (45/96)	WES changed medical management and impacted family planning in 45/96	Flag 1 - main analysis	Mixed NDD	Full-text verified: "WES results changed medical management and impacted family planning for DD/ID children (45/96)". 96/225=42.67% (95% CI

Study	Design	Population (N, Age, Diagnosis)	Method	Dominant Variant Type	Yield %	Action. %	Key Outcome	Sens. Flag	NDD Category	Flag Notes / Denominator Decisions
							diagnosed (22 reproductive decision changes, 5 disease-monitoring initiations)			36.15–49.18%) confirmed exact from Results.
Monroe et al., 2016	Pilot cohort (random selection)	17 children; ID, Netherlands	Trio-WES	Diagnostic variants	29.4% (5/17)	— (yield only)	Average cost of the traditional diagnostic trajectory ($16,409 per patient) exceeded trio-WES cost ($3,972); trio-WES avoided an average of $3,547 in genetic and metabolic investigations in diagnosed patients	Flag 1 - main analysis	ID	No formal actionability fraction reported (cost-effectiveness focus). Full-text verified: "Diagnostic variants were found in 5 of the 17 patients… 29.4%". Patients were randomly selected for this pilot study; authors acknowledge the sample size (n = 17) is limited. Primary purpose was cost comparison, not prevalence estimation.
Ilic et al., 2024	Retrospective cohort	100 children (2 centres); DD/ID	WES	Diverse pathogenic variants	66.0% (66/100)	— (yield only)††	Reverse phenotyping (RP) was indicated in 33/100 patients after WES (half of the patients with positive WES findings)	Flag 1 - main analysis	Mixed NDD	No formal actionability fraction reported; RP data discussed narratively (Section 4). Full-text verified: yield consistent between the two centres (66.1% vs. 65.9%). NOTE: internal inconsistency identified between Section 2.1 (Ljubljana/Barcelona as testing sites) and Section 2.2 (Serbia/Bosnia as data-source institutions) of the original article - reflected in JBI Q1 rating (Table 1).

Abbreviations: ASD=Autism Spectrum Disorder; DD-ID=Developmental Delay/Intellectual Disability; DEE=Developmental & Epileptic Encephalopathy; GDD=Global Developmental Delay; CMA=Chromosomal Microarray; WES=Whole-Exome Sequencing; WGS=Whole-Genome Sequencing; CNV=Copy Number Variant; VUS=Variant of Uncertain Significance. § Nakhleh Francis 2023: actionability reflects the specialist-referral component of a non-aggregable multi-category framework (see Table S1). ¶ Charouf 2024: actionability counts etiology-specific treatment changes (medication or dietary) only, reported combined across WES and WGS arms and not separable by modality; specialist referrals (15 patients) are not included (see Table S1). †† Ilic 2024: reverse phenotyping (RP) data discussed narratively, not a formal actionability fraction.

Table S3. Summary of Findings and Genotype-Directed Clinical Actions

Referenced in the main text at Section 3.3.

The full expansion of the genotype-directed clinical actions underlying Table 2.

Study	Genetic Finding / CNV	Clinical Action Taken	Impact on Management
Xu 2018	Recurrent & non-recurrent CNVs (e.g., 7q11.23, 15q11-q13, 22q11.21, 17q11.2, NRXN1, SHANK3)	Multidisciplinary referrals, specialized metabolic/endocrine/cardiac testing, EEG, medication adjustments, and prenatal counseling	Enabled earlier diagnosis (61%), improved prognosis (50.8%), medication optimization, and actionable care in 94.9% of positive cases
Jang 2019	Pathogenic variants (e.g., 16p11.2 microdeletion, Prader-Willi syndrome, Down syndrome, Duchenne muscular dystrophy) and variants of possible significance (VPS)	Multidisciplinary specialist referrals (neurology, cardiology, endocrinology, musculodystrophy clinics), advanced diagnostic imaging (brain CT/MRI, skeletal surveys), and targeted pharmacologic management (hormonal therapies, dietary restrictions, seizure prophylaxis)	Directly modified clinical strategies in 83.3% of pathogenic and 86% of VPS cases; facilitated earlier care, personalized developmental surveillance, avoided unnecessary procedures, and shortened the diagnostic odyssey
Henderson 2014	FBN1, CTNS, SHOX, 18q/18p deletions, Klinefelter, APC, VHL, MLH1, NSD1, SMARCB1, CREBBP, 4q21, mosaic monosomy 7, Williams syndrome, 22q11.2 deletion	Pharmacologic therapy (ARBs, cysteamine, growth hormone, testosterone); cancer surveillance/screening (ultrasounds, alpha-fetoprotein, oncology consultations, bone marrow transplant); contraindications (avoiding calcium/vitamin D, live viral vaccines)	Normalized aortic root growth; preserved renal function; maximized growth potential; enabled early detection/prevention of hereditary cancers; averted metabolic and vaccine-related complications
Coulter 2011	Diverse abnormal CNVs (e.g., 3.8-Mb 2q/PROC deletion, 244-kb 22q12.1/CHEK2 deletion, Xp21.1/DMD deletion, 16p11.2 microdeletions/duplications) and VPS	Targeted specialist referrals (neurology, oncology, cardiology, endocrinology), diagnostic imaging (MRI, ultrasound), and specific laboratory tests (e.g., creatine kinase, protein C antigen)	Facilitated condition-specific medical surveillance (contraindicated drugs, cancer screening, cardiac monitoring), provided definitive diagnostic clarity, reduced redundant work-ups
Saam 2008	Known syndromes (Sotos, DMD, 1p36, PWS) and novel copy number alterations (deletions/duplications)	Recurrence risk counseling; specialist referrals (cardiology, endocrinology) and medical screenings (kidney/thyroid); educational/insurance services; avoided redundant diagnostic/genetic testing	70.8% of patients experienced management changes, resulting in targeted medical care, optimized diagnostic pathways, and improved family support via diagnostic certainty
Demos 2019	Diverse (e.g., SCN1A, KCNQ2, ATP1A3, ALDH7A1, POLG, STXBP1)	Discontinued contraindicated anti-seizure medications (sodium channel blockers for SCN1A), initiated precision therapies (stiripentol, flunarizine for ATP1A3, B6/lysine-restricted diet for ALDH7A1), targeted genetic/prenatal counseling	Optimized seizure control, reduced seizure/hemiplegic episode frequency, prevented medication-induced toxicity (e.g., valproic acid liver toxicity), informed long-term clinical and reproductive decision-making
Selvanayagam 2025*	Diverse variants (e.g., SHANK3, ARID1B, GRIN2B, ASH1L, SCN2A)	Multidisciplinary assessments; targeted symptom screening; research participation (e.g., iPSC studies)	Enabled personalized medical management, ended diagnostic odysseys, facilitated targeted therapeutic research and family support
Harris 2020	Pathogenic CMA: 15q/22q deletions/duplications, 48 XXYY, mosaic trisomy 8, various small deletions. Fragile X: full mutations, premutations, "grey zone," and mosaicism	Referrals to subspecialists (endocrinology, metabolism, sleep medicine), echocardiograms, renal ultrasounds, audiology/ophthalmology exams, seizure monitoring, Fragile X clinic referral	72.2% of pathogenic cases prompted specific medical recommendations, informing ongoing clinical care and monitoring beyond initial diagnosis
Tao 2014	Pathogenic/likely pathogenic CNVs (1p36 deletion, 22q11.2, Williams syndrome, Prader-Willi, Wolf-Hirschhorn)	28/37 (75.7%) cases prompted actions: 24 referrals, 25 diagnostic tests, 19 surveillance checks, 7 procedures, 15 medication adjustments, 12 lifestyle modifications	Enabled evidence-based personalized medical management, avoided unnecessary investigations, ended the diagnostic odyssey for affected patients
Ji 2019	Pathogenic variants (e.g., XIAP, MTM1, KCNQ1) and cancer-predisposition genes (ATRX, RAD50, MEN1, RET, CHEK2)	Targeted interventions including allogeneic hematopoietic cell transplantation (HSCT), G-tube placement, oncology surveillance, cardiology referral	Enabled curative therapy, averted ineffective treatments, facilitated proactive risk management, allowed early-stage, curable cancer detection
Pagan 2017†	Rare non-synonymous AANAT/ASMT variants and recurrent ASMT microduplication (exons 1-7); no statistically significant enrichment in ASD vs. controls ($p=0.56$; $p=0.48$)	None documented for these specific genetic findings; research focused on biochemical characterization rather than clinical intervention based on genotype	Genetic findings did not necessitate a change in clinical management. Findings suggest melatonin as a therapeutic target for ASD-associated sleep disorders regardless of specific genetic status
Stavropoulos 2016	Diverse pathogenic CNVs (22q11.2 deletion, 8q22.1, 16p13.11, ChrX deletion/Turner) and primary diagnostic SNVs/indels (CBL, ATP1A3, NGLY1, KAT6B, COL4A1); medically actionable secondary findings in SCN5A, FBN1, KCNH2, RYR2, DSG2	Genetic counselling for all diagnostic findings; specialized clinic referrals (e.g., 22q11.2 deletion clinic); tailored surveillance protocols (e.g., leukaemia monitoring for CBL); corrected clinical trajectories; cascade testing of at-risk relatives	38% of patients benefited from genetic counselling when primary + medically actionable secondary findings combined; informed disease-specific monitoring and family-level risk management
Du 2018	Pathogenic variants (AFF2, IL1RAPL1, POGZ, ADNP)	Targeted systemic screenings (endocrine, gastrointestinal, neurological, skeletal, eye, ear); initiated growth hormone therapy;	Enabled precise, personalized therapeutic interventions and systemic workups;

Study	Genetic Finding / CNV	Clinical Action Taken	Impact on Management
		discontinued brain protein hydrolysate; genetic counseling for future pregnancies	facilitated cessation of ineffective treatments; informed reproductive planning
Minardi 2020	CACNA1E, SCN1A, ANKRD11 (KBG), DYRK1A, ZBTB20, and family-specific variants	Targeted therapy (e.g., Topiramate), avoided contraindicated drugs (sodium channel blockers), tapered antiseizure medications in seizure-free cases, planned follow-up screening, genetic counseling	Optimized personalized care through targeted therapeutic adjustments, prevention of inappropriate pharmacological treatment, proactive comorbidity management, informed reproductive planning
Wu et al., 2023	Recurrent microdeletion/duplication syndromes: Williams-Beuren (n = 4), Angelman/Prader-Willi (n = 9), DiGeorge, 2q37 deletion, Waardenburg, plus rare deletions at 5q33.2q34 and 5q14.3q15 and other CNVs	Not formally quantified as a management fraction in source article	CMA yield (37.7%) enabled recognition of multiple clinically established microdeletion/duplication syndromes across a phenotypically heterogeneous DD/ID cohort; no formal actionability fraction reported
Cucinotta et al., 2023	Pathogenic/likely pathogenic CNVs in ASD cohort (+27.1% VUS)	Additional medical investigations prescribed in 13/50 P/LP cases (25 total exams, mainly brain MRI, EEG, EKG, and/or cardiac ultrasound)	12/25 (48.0%) additional investigations yielded a positive finding, directly informing further clinical management
Nakhleh Francis et al., 2023	Diverse diagnostic variants in a publicly funded GDD testing program	Specialist referral (27% of diagnosed), new surveillance recommendations (19%), family-focused outcomes (23%), active management change (9%) - categories not mutually exclusive	Informed clinical pathway across multiple non-aggregable domains; referral component used as primary quantitative estimate (Section 2.3/2.4)
Hakizimana et al., 2026	Likely pathogenic: SNVs in GABRB3 and SYNGAP1 and CNVs at the GNAS locus and 1p35.3-p35.2; phenotype-concordant VUS in SYNJ1, KIF15, SHANK3, HUWE1 and EPG5	Not formally quantified as a management fraction in source article	4/31 (12.9%) likely pathogenic findings (29.0% including phenotype-concordant VUS), predominantly in syndromic ASD with comorbid ID/epilepsy/GDD; demonstrated feasibility of trio-WES with NDD-focused gene panel in a resource-limited African clinical genetics setting
Charouf et al., 2024	Diverse variants (including FOLR1, QDPR, GRIN2B, SCN1A) in epilepsy+NDD cohort	Major, potentially life-saving medical management changes (medication adjustment, dietary intervention) in 6 children; in addition, 15 patients required specialist referral for screening and surveillance of non-CNS comorbidities, and pre-pregnancy counselling was performed in 23 families with inherited disease (not counted in the actionability fraction)	6/31 (19.4%) of diagnosed children had documented clinical improvement attributable to genotype-directed management change
Abarca-Barriga et al., 2025	MECP2 (most frequent, 12.8% of positive cases - Rett syndrome spectrum), LAMA2 (second most frequent, 7.7% - congenital muscular dystrophy spectrum), STXBP1 (third, 5.1% - developmental and epileptic encephalopathy type 4); second-tier CMA positive in 2/10 WES-negative patients	Not formally quantified as a management fraction in source article	Demonstrated WES diagnostic value in a Peruvian paediatric ID population, with MECP2/LAMA2/STXBP1 as the three most frequent contributory genes; informed second-tier testing strategy (CMA after negative WES)
Ma et al., 2024	Diverse pathogenic variants in DD/ID cohort (208/225 trio-based)	Medical management changes and reproductive planning impact: 22 reproductive decision changes, 5 disease-monitoring initiations (among other actions, 45/96 total)	45/96 (46.9%) of diagnosed patients had WES-informed changes to medical management and/or family planning
Monroe et al., 2016	CTNNB1, ANKRD11 (KBG syndrome), ADNP, SMARCB1, CHD4 - causal in 5/5 diagnosed patients; further delineated CTNNB1 haploinsufficiency and KBG syndrome phenotypes	Not formally quantified as a management fraction in source article (cost-effectiveness focus)	Trio-WES cost $3,972 per patient versus $16,409 for the traditional diagnostic trajectory (average duration 6.6 years), with average savings of $3,547 in genetic and metabolic investigations for diagnosed patients; all five causal genes had been known to be causal for less than 4 years, reflecting the rapid pace of gene discovery
Ilic et al., 2024	Diverse pathogenic variants in DD/ID cohort across two clinical centres	Reverse phenotyping (RP): systematic clinical re-evaluation triggered by WES-positive results	RP indicated in 33/100 patients after WES (half of the patients with positive WES findings), refining phenotype-genotype correlation and clarifying WES's optimal place in the diagnostic sequence (discussed narratively, Section 4)

* Selvanayagam et al. (2025): actionability reflects broad 3-category framework (medical management + reproductive counselling + psychosocial support) - not directly comparable to other studies (see Section 2.4). † Pagan et al. (2017): included for qualitative narrative synthesis only; excluded from quantitative meta-analysis (p=0.56 vs. controls; no actionability documented) - see Section 2.3.

Table S4. GRADE Summary of Findings for Diagnostic Yield and Clinical Actionability

Certainty of evidence assessed using the GRADE approach, adapted for single-group prevalence/proportion meta-analyses in the absence of a comparator group (see Section 2.5 and 3.2 of the main text).

Domain	Rating	Downgrade	Rationale
Outcome: Diagnostic Yield			
Study design	Observational (single-arm proportion)	Start: Low	No comparator group; 23 data points from 22 studies, N = 7,641
Risk of bias	Serious	−1	JBI (Prevalence checklist) mean 7.18/9: 17/22 High, 4/22 Moderate (Xu 2018, Henderson 2014, Charouf 2024, Ilic 2024), 1/22 Low (Saam 2008); sample-size justification (Q3) unmet in all 22 studies
Inconsistency	Very serious	−2	I^2 = 96.1%; pooled estimate 28% (95% CI 21–35%); persists across sensitivity analyses (flag=1: 24%, 95% CI 16–33%) and modality subgroups (CMA 16%, WES 36%, WGS 43%; Q-between χ^2=24.73, p<0.0001)
Indirectness	Serious	−1	Non-standard yield definition in Selvanayagam et al. (2025) (research return-of-results, not standard clinical yield)
Imprecision	Serious	−1	Wide 95% CI relative to point estimate (21–35% around 28%); 95% prediction interval even wider (2–67%), reflecting extreme heterogeneity
Publication bias	Suspected, scored	−1	Egger's test p = 0.0008 (t=3.94, df=21); asymmetry judged attributable to structural heterogeneity (WGS/WES studies smaller and higher-yield) rather than selective reporting
Overall certainty	Very Low		(no additional row content)
Outcome: Clinical Actionability			
Study design	Observational (single-arm proportion)	Start: Low	No comparator group; 16 studies, N = 1,538
Risk of bias	Serious	−1	Same JBI distribution as above
Inconsistency	Very serious	−2	I^2 = 95.7%; pooled estimate 58% (95% CI 44–72%); range across sensitivity analyses 54–64%
Indirectness	Serious	−1	Non-standard denominators (Jang 2019; Saam 2008; Henderson 2014; Nakhleh Francis 2023 - referral-only component); broader three-category framework in Selvanayagam et al. (2025); combined non-separable modality in Charouf et al. (2024)
Imprecision	Serious	−1	Very wide 95% CI (44–72%); WGS subgroup CI uninterpretable (6–100%, k=2); 95% prediction interval 5–100%

Domain	Rating	Downgrade	Rationale
Publication bias	Undetected	-	Egger's test p = 0.3307 (t=-1.01, df=14); no significant asymmetry
Overall certainty	Very Low		(no additional row content)

Table S5. Full Search Strategies for PubMed and Web of Science

Full, verbatim search strings, filters, and dates of execution for both databases (see Section 2.1 of the main text).

Database	Full search string (verbatim)	Filters applied	Date executed	Records retrieved
PubMed	("genetic testing" OR "genomic testing" OR "WES" OR "Whole Exome Sequencing" OR "exome sequencing" OR "genome sequencing" OR "whole genome sequencing" OR "WGS" OR "microarray")<br>AND<br>("autism" OR "ASD" OR "Autism Spectrum Disorder" OR "ADHD" OR "Attention-Deficit/Hyperactivity Disorder" OR "language delay" OR "Neurodevelopmental Disorders" OR "NDD" OR "intellectual disability" OR "developmental delay" OR "global developmental delay")<br>AND<br>("personalized medicine" OR "individualized therapy" OR "clinical management" OR "therapeutic management" OR "clinical utility" OR "clinical impact" OR "actionability")	Abstract; Free full text; Full text; English; 2015/11/20–2026/10/01	01 October 2026	n = 335
Web of Science	Same three concept blocks as above, run as Web of Science Smart Search (semantic), applied in 3 sequential refinement steps (one concept block per step).	Publication Years 2015–2026; Languages: English; Document Types: Article; Open Access: All Open Access	01 October 2026	n = 199

Table S6. Studies Excluded at Full-Text Review, with Reasons

Of the reports assessed for eligibility at full-text review, 14 were excluded; reasons are grouped into the four categories reported in the main text, with three records excluded for population scope outside the NDD definition used in this review (epilepsy-only cohorts without comorbid ID, ASD, or GDD).

Excluded study	Study type / method	Reason for exclusion
McKnight et al., 2022	Retrospective cohort	Population outside NDD scope (epilepsy-only cohort; no comorbid ID/ASD/GDD inclusion criterion)
Koh et al., 2023	Retrospective cohort	Population outside NDD scope (epilepsy-only cohort; no comorbid ID/ASD/GDD inclusion criterion)
Wu et al., 2026	Retrospective cohort	Population outside NDD scope (epilepsy-only cohort; no comorbid ID/ASD/GDD inclusion criterion)
Besterman et al., 2025	Case reports (3 cases)	Case reports with n < 5
Reiff et al., 2015	Retrospective parent-perception study (mixed methods)	Absence of quantitative yield/actionability data
Stafford & Sanchez-Lara, 2022	Review	Review/guideline without primary data
Hyman et al., 2022	Clinical report (AAP guideline update)	Review/guideline without primary data
Baker & Jeste, 2015	Review	Review/guideline without primary data
Savatt & Myers, 2021	Review	Review/guideline without primary data
Kreiman et al., 2020	Review article (includes 5 illustrative case reports)	Review/guideline without primary data
Collins et al., 2015	Perspective piece	Review/guideline without primary data
Johannessen et al., 2017	Questionnaire-based survey of parental attitudes	Absence of quantitative yield/actionability data
Butler et al., 2022	Case reports (3 cases)	Case reports with n < 5
Meral et al., 2024	Narrative review	Review/guideline without primary data

Figure S1. Forest Plot - Diagnostic Yield

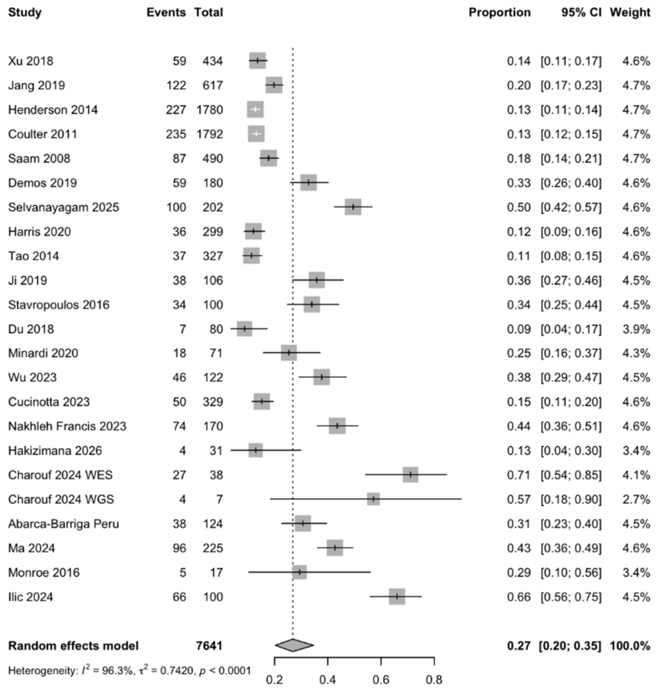

Figure S1. *Forest plot of pooled diagnostic yield across 23 data points from 22 studies using logit transformation (sensitivity analysis). Random-effects model, REML estimation. Pooled estimate: 26.84% (95% CI 20.25–34.65%), consistent with the primary Freeman–Tukey estimate (28%, 95% CI 21–35%) and the GLMM estimate (26.63%, 95% CI 20.15–34.31%; not shown graphically - see Table S1).*

Figure S2. Forest Plot - Clinical Actionability

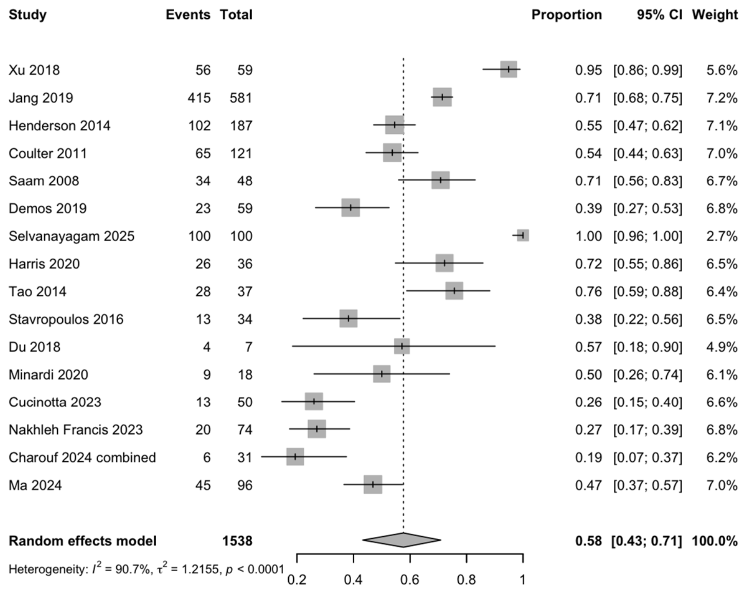

Figure S2. Forest plot of pooled clinical actionability across 16 studies using logit transformation (sensitivity analysis). Random-effects model, REML estimation. Pooled estimate: 57.66% (95% CI 43.26–70.86%), consistent with the primary Freeman–Tukey estimate (58%, 95% CI 44–72%) and the GLMM estimate (61.12%, 95% CI 43.36–76.35%; not shown graphically - see Table S1).

Figure S3. Forest Plot with Prediction Interval - Diagnostic Yield

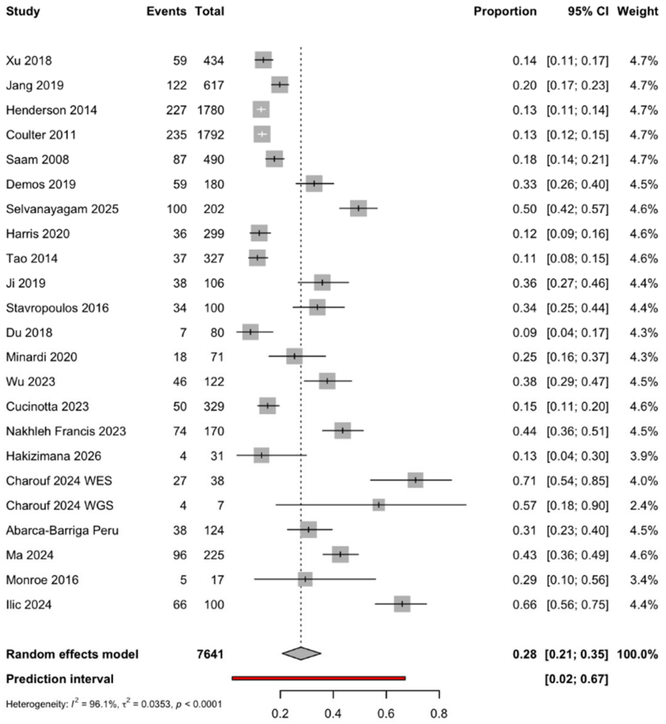

Figure S3. Forest plot of pooled diagnostic yield across 23 data points from 22 studies with 95% prediction interval. The prediction interval (2–67%) is substantially wider than the 95% confidence interval (21–35%), reflecting the high between-study heterogeneity (I^2 = 96.1%) and indicating the range of yield plausible in a new clinical setting.

Figure S4. Forest Plot with Prediction Interval - Clinical Actionability

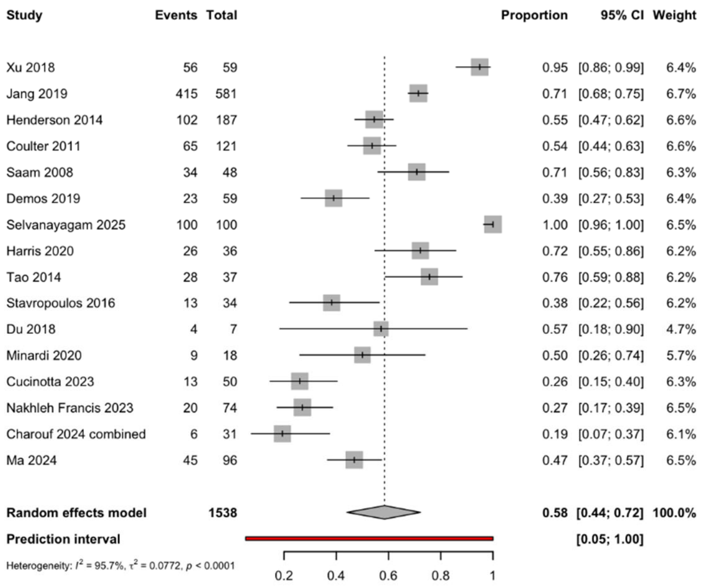

Figure S4. Forest plot of pooled clinical actionability across 16 studies with 95% prediction interval. The prediction interval (5–100%) is substantially wider than the 95% confidence interval (44–72%), reflecting the high between-study heterogeneity (I^2 = 95.7%) and indicating the range of actionability plausible in a new clinical setting.
